# Applicability of guideline-informing lipid-lowering clinical trials to contemporary real-world patients with atherosclerotic cardiovascular disease

**DOI:** 10.1093/ehjqcco/qcaf091

**Published:** 2025-08-29

**Authors:** Pauline C E Schuitema, Manon C Stam-Slob, Frank L J Visseren, Charlotte Koopal, the UCC-SMART Study Group, M J Cramer, M G van der Meer, P van der Harst, H M Nathoe, J van Setten, N C Onland-Moret, M van Smeden, M H Emmelot-Vonk, P A de Jong, A T Lely, S Haitjema, M M Mokhles, Y M Ruigrok, M C Verhaar, J A N Dorresteijn, F L J Visseren

**Affiliations:** Department of Vascular Medicine, University Medical Center Utrecht, Utrecht University, Heidelberglaan 100, 3584 CX Utrecht, The Netherlands; Department of Vascular Medicine, University Medical Center Utrecht, Utrecht University, Heidelberglaan 100, 3584 CX Utrecht, The Netherlands; Department of Vascular Medicine, University Medical Center Utrecht, Utrecht University, Heidelberglaan 100, 3584 CX Utrecht, The Netherlands; Department of Vascular Medicine, University Medical Center Utrecht, Utrecht University, Heidelberglaan 100, 3584 CX Utrecht, The Netherlands

**Keywords:** Atherosclerotic cardiovascular disease, Lipid-lowering therapy, Randomized clinical trials, Trial eligibility, Real-world evidence, Secondary prevention

## Abstract

**Aims:**

Lipid-lowering randomized clinical trials (RCTs) inform guideline recommendations for secondary prevention of atherosclerotic cardiovascular disease (ASCVD). However, these trials were often conducted decades ago using strict eligibility criteria, which may limit their applicability to today’s diverse ASCVD population. This study compared clinical characteristics and long-term outcomes between trial-eligible and ineligible real-world ASCVD patients.

**Methods and results:**

Eligibility criteria from eight pivotal lipid-lowering RCTs were applied to patients with coronary artery disease (CAD), cerebrovascular disease (CeVD), and peripheral artery disease (PAD) in the Utrecht Cardiovascular Cohort–Second Manifestations of ARTerial disease (UCC-SMART) study (2000–2023). The proportion of patients meeting eligibility criteria was determined for each trial. Differences in clinical characteristics between trial-eligible and ineligible patients were assessed, and risks of recurrent cardiovascular (CV) events and all-cause mortality were compared using cumulative incidence curves with 95% confidence intervals, accounting for competing risks. Among 8537 patients with ASCVD (5673 CAD; 2493 CeVD; 1302 PAD), median follow-up was 8.8 years (interquartile range: 4.2–13.9). Trial eligibility ranged from 9 to 85% for CAD, 7 to 42% for CeVD, and 8 to 78% for PAD. Eligibility was generally higher for earlier trials. Trial-eligible and ineligible patients differed in clinical characteristics in most trials, but their risks of recurrent CV events and all-cause mortality were largely comparable.

**Conclusion:**

Eligibility of real-world ASCVD patients for lipid-lowering RCTs varied substantially across trials and ASCVD subtypes. Despite differences in clinical characteristics, trial-eligible and ineligible patients generally had comparable outcomes, suggesting that guideline-informing lipid-lowering trials are broadly representative of contemporary real-world ASCVD patients.

## Graphical Abstract

**Graphical Abstract:**
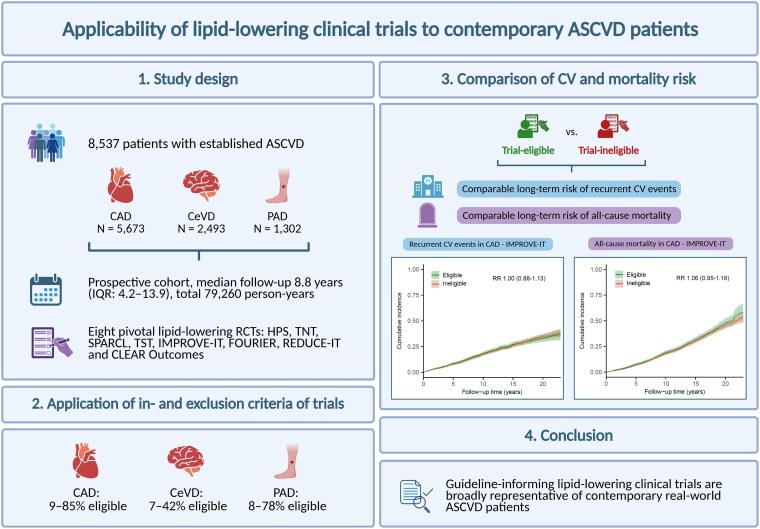

Key Learning Points What is already known:

- Lipid-lowering randomized clinical trials (RCTs) form the foundation of guideline recommendations for secondary atherosclerotic cardiovascular disease (ASCVD) prevention.
- Many of these trials were conducted decades ago using strict eligibility criteria, which may limit their applicability to the diverse population of contemporary real-world ASCVD patients.
- Common comorbidities and key demographic groups are often underrepresented in lipid-lowering RCTs.

What this study adds:

- Eligibility for lipid-lowering RCTs varies widely across trials and ASCVD subtypes, with earlier trials generally demonstrating higher eligibility rates than more recent ones.
- Despite differences in clinical characteristics, trial-eligible and ineligible patients exhibit largely comparable long-term risks of recurrent cardiovascular events and all-cause mortality.
- These findings suggest that guideline-informing lipid-lowering trials are broadly representative of contemporary real-world patients with ASCVD, reinforcing their continued relevance for clinical guidelines.

## Introduction

Lipid-lowering randomized clinical trials (RCTs) inform guideline recommendations for lipid-lowering therapy (LLT) in secondary prevention of atherosclerotic cardiovascular disease (ASCVD) in patients with coronary artery disease (CAD), cerebrovascular disease (CeVD), and peripheral artery disease (PAD).^1-9^ These trials often use strict eligibility criteria to enhance internal validity by selecting a more homogeneous study population and ensuring that treatment effects reflect true causal relationships. However, such criteria may exclude a substantial proportion of contemporary real-world patients, potentially limiting the applicability of trial findings to routine clinical practice.^10^

Many lipid-lowering RCTs exclude patients with common comorbidities, such as kidney disease, heart failure, immunosuppressant conditions, or cancer.^11^ In addition, key demographic groups, including women and older adults, are often underrepresented.^11^ It is unknown what proportion of contemporary real-world patients with CAD, CeVD, and PAD would meet eligibility criteria for pivotal lipid-lowering trials, which form the basis of current clinical guideline recommendations. Also, it is unclear whether trial-eligible and ineligible patients differ substantially in clinical characteristics and long-term prognosis.

The cardiovascular (CV) risk profile of contemporary ASCVD patients may have changed significantly since the publication of lipid-lowering RCTs due to advances in vascular treatments, updated clinical guidelines, greater availability of generic preventive medications, and improved management of cardiometabolic comorbidities. Over the past few decades, there has been a notable reduction in both hospital admissions and mortality rates associated with CV events.^12,13^ If these improvements in CV risk profiles would have led to the exclusion of patients from pivotal lipid-lowering trials conducted in the 1990s, caution is warranted when extrapolating those trial findings to today’s clinical populations.

The present study assessed the proportion of contemporary real-world patients with CAD, CeVD, and PAD meeting eligibility criteria for pivotal lipid-lowering RCTs and compared clinical characteristics and long-term risks of recurrent CV events and all-cause mortality between trial-eligible and ineligible patients.

## Methods

### Trial selection

Lipid-lowering RCTs informing treatment recommendations for secondary ASCVD prevention in CAD, CeVD, and PAD patients were identified from European and American guidelines.^1-9^ This was done by systematically reviewing the references cited in these guidelines. When multiple studies or meta-analyses supported a recommendation, we pragmatically selected the largest trial for each lipid-lowering agent in CAD, CeVD, and PAD populations. If two trials were of comparable size but addressed different domains, the trial with the broader scope was chosen. For statin therapy, one trial was selected comparing statin therapy to placebo, and another comparing more vs. less intensive statin treatment.

When applying these criteria, the Heart Protection Study (HPS) and the Treating to New Targets (TNT) trial were selected for statin therapy in CAD and PAD populations, while the Stroke Prevention by Aggressive Reduction in Cholesterol Levels (SPARCL) study and the Treat Stroke to Target (TST) trial were selected for statin therapy in CeVD patients.^14-17^ Additionally, for CAD, CeVD, and PAD, the IMProved Reduction of Outcomes: Vytorin Efficacy International Trial (IMPROVE-IT) was selected for ezetimibe (added to statin), the Further Cardiovascular Outcomes Research with PCSK9 Inhibition in Subjects with Elevated Risk (FOURIER) trial for proprotein convertase subtilisin/kexin type 9 (PCSK9) inhibition, the Reduction of Cardiovascular Events with Icosapent Ethyl–Intervention trial (REDUCE-IT) for icosapent ethyl, and the Cholesterol Lowering via Bempedoic Acid, an ACL-Inhibiting Regimen (CLEAR) Outcomes trial for bempedoic acid.^18-21^

### Operationalization of trial eligibility criteria

Eligibility criteria for each trial were extracted from original trial protocols and related publications. An overview of these criteria and their operationalization in this study is provided in Supplementary material online, *Table S1*. Given that LLT is now standard care for ASCVD patients, some trial criteria were adjusted to reflect current clinical practice. For instance, HPS excluded patients with a statin treatment indication, and SPARCL excluded those receiving any LLT at baseline; these criteria were not applied in the analysis. Additionally, several trials defined eligibility criteria based on untreated lipid levels. To approximate these in our study, lipid values were adjusted according to each trial’s specifications. For HPS, total cholesterol values were recalculated assuming a 26% reduction with statin therapy, based on reported reductions ranging from 17 to 35% across various statins and doses.^22^ For SPARCL, LDL cholesterol (LDL-C) values were adjusted assuming a 30% reduction from LLT, based on reported reductions ranging from 24 to 49% across various statins and doses.^22^ For TNT, where participants underwent an 8-week run-in period with atorvastatin 10 mg, LDL-C values for patients not on LLT were recalculated assuming a 37% reduction with atorvastatin 10 mg.^22,23^ CLEAR Outcomes required statin intolerance for inclusion, which was operationalized in our study by excluding all patients using statins at baseline.

### Study population

Eligibility criteria from the eight selected lipid-lowering trials were applied to patients with established CAD, CeVD, and PAD enrolled in the Utrecht Cardiovascular Cohort–Second Manifestations of Arterial Disease (UCC-SMART) study between January 2000 and January 2023. The UCC-SMART study is an ongoing prospective cohort of individuals aged 18–90 years with high CV risk who were referred to a Dutch teaching hospital. The study was approved by the local Medical Research Ethics Committee, and all participants provided written informed consent. A comprehensive description of the study design and methodology has been published previously.^24^

ASCVD subtypes were defined based on the original UCC-SMART protocol: CAD included prior myocardial infarction, angina pectoris, ≥1 vessel disease on coronary angiography, percutaneous coronary intervention, or coronary artery bypass grafting; CeVD included transient ischaemic attack, cerebral infarction, ischaemic retinal syndrome, carotid surgery, or angioplasty; and PAD included Fontaine stage ≥2, amputation, vascular surgery, or angioplasty.^24^

### Baseline data collection and outcome assessment

At enrolment, participants underwent standardized vascular screening, including health questionnaires, physical examinations, and laboratory testing.^24^ During follow-up, annual questionnaires were administered to assess outcome events. When a potential CV event or mortality was reported, additional information was obtained from hospital and general practice records and independently reviewed by three members of the UCC-SMART endpoint committee.

The primary outcome was recurrent CV events, including myocardial infarction, stroke, and CV mortality. The secondary outcome was all-cause mortality. All outcomes were defined according to the original study protocol.^24^ Patients were followed from enrolment until the occurrence of a CV event, death, loss to follow-up, or 1 January 2023 (the last update of UCC-SMART outcome data).

### Data analyses

The proportion of UCC-SMART patients eligible for each trial was determined by applying the respective inclusion and exclusion criteria. Missing data ranged from 0.01% for diabetes history to 23.9% for glycated haemoglobin (HbA1c) and were addressed using multiple imputation with 40 imputations and 40 iterations (mice package, RStudio).^25^ Convergence was verified through trace plots. Clinical characteristics were described separately for trial-eligible and ineligible patients within each ASCVD subtype and trial. Differences between trial-eligible and ineligible patients were assessed using pooled *t*-tests for normally distributed variables, pooled Wilcoxon rank-sum tests for skewed variables, and pooled χ² tests for categorical variables. The 10-year predicted absolute risk of recurrent ASCVD was estimated using the Secondary Manifestations of ARTerial disease (SMART2) risk score.^26^ Differences in long-term prognosis between trial-eligible and ineligible patients were evaluated using cumulative incidence curves with 95% confidence intervals (CIs), accounting for non-CV deaths as competing events for the primary outcome. Patients were censored at the time of death or loss to follow-up, whichever came first. Additionally, incidence rates per 100 person-years and pooled rate ratios with 95% CIs for trial-eligible vs. trial-ineligible patients were calculated. A *P*-value <0.05 was considered statistically significant. All analyses were conducted using RStudio version 4.3.1 (R Foundation for Statistical Computing, Vienna, Austria).

### Sensitivity analysis

To assess whether results differed in a more contemporary subset of the study population, a sensitivity analysis was conducted in patients included from 2010 onward.

## Results

The study included 8537 patients with established ASCVD, of whom 5673 (66%) had CAD, 2493 (29%) had CeVD, and 1302 (15%) had PAD. The mean age was 61 ± 10 years, 73% were male, and 14% had multiple ASCVD manifestations. The median follow-up was 8.8 years (interquartile range: 4.2–13.9 years) and the total follow-up was 79260 person-years. During follow-up, recurrent CV events occurred in 1104 (19%) CAD patients, 479 (19%) CeVD patients, and 317 (24%) PAD patients. All-cause mortality was recorded in 1309 (23%) CAD patients, 655 (26%) CeVD patients, and 515 (40%) PAD patients.

### Proportion of trial-eligible patients

**Figure 1:**
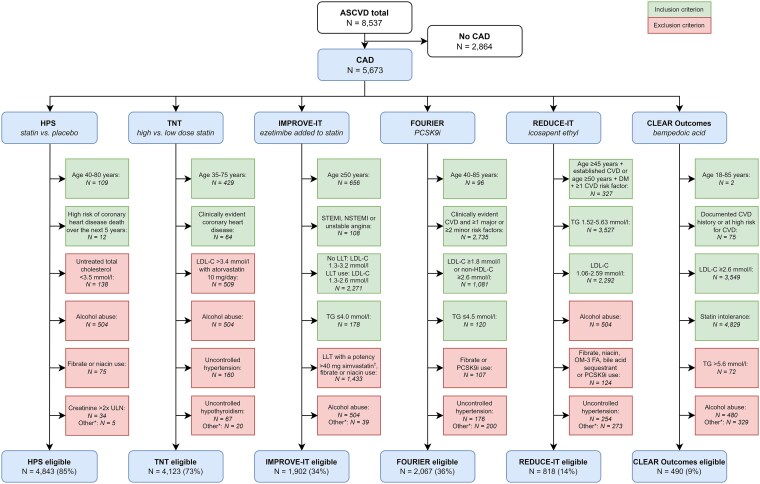
Application of trial eligibility criteria to UCC-SMART patients with coronary artery disease. Numbers in rectangles represent the number of patients not fulfilling that eligibility criterion. UCC-SMART, Utrecht Cardiovascular Cohort–Second Manifestations of ARTerial disease; ASCVD, atherosclerotic cardiovascular disease; N, number; CAD, coronary artery disease; HPS, Heart Protection Study; TNT, Treating to New Targets trial; IMPROVE-IT, IMProved Reduction of Outcomes: Vytorin Efficacy Intervention Trial; FOURIER, Further Cardiovascular Outcomes Research with PCSK9 Inhibition in Subjects with Elevated Risk; PCSK9i, proprotein convertase subtilisin/kexin type 9 inhibitor; REDUCE-IT, Reduction of Cardiovascular Events with Icosapent Ethyl–Intervention Trial; CLEAR Outcomes, Cholesterol Lowering via Bempedoic Acid, an ACL-Inhibiting Regimen Outcomes trial; ULN, upper limit of normal; LDL-C, low-density lipoprotein cholesterol; STEMI, ST-elevation myocardial infarction; NSTEMI, non-ST-elevation myocardial infarction; LLT, lipid-lowering therapy; TG, triglycerides; non-HDL-C, non-high-density lipoprotein cholesterol; DM, diabetes mellitus; OM-3 FA, omega-3 fatty acid. * Eligibility criteria under ‘Other’ varied between trials. HPS: concurrent cyclosporin use. TNT: HbA1c > 10%. IMPROVE-IT: creatinine clearance <30 mL/min and concomitant medications. FOURIER: known haemorrhagic stroke at any time, TSH < LLN or >1.5× ULN and estimated glomerular filtrate rate <20 mL/min/1.73 m^2^. REDUCE-IT: HbA1c > 10%, creatinine clearance <30 mL/min and concomitant medications. CLEAR Outcomes: estimated glomerular filtrate rate <30 mL/min/1.73 m^2^, SBP ≥180 mmHg or DBP ≥110 mmHg, HbA1c ≥ 10%, TSH >1.5× ULN and haemoglobin <10 g/dL. † In the trial protocol defined as simvastatin >40 mg, atorvastatin ≥40 mg, all doses of rosuvastatin and ezetimibe co-administered with any dose of any statin.

Among CAD patients, the majority were eligible for HPS (85%) and TNT (73%) (*Figure 1*). Approximately one-third met the eligibility criteria for IMPROVE-IT (34%) and FOURIER (36%), while only a minority qualified for REDUCE-IT (14%) and CLEAR Outcomes (9%).

Among CeVD patients, 42% met the criteria for TST, 38% for SPARCL, and 31% for FOURIER (see Supplementary material online, *Figure S1*). Eligibility was lower for CLEAR Outcomes (10%), REDUCE-IT (8%), and IMPROVE-IT (7%).

Among PAD patients, the majority were eligible for HPS (78%) and FOURIER (65%), with nearly one-quarter qualifying for CLEAR Outcomes (24%) (see Supplementary material online, *Figure S2*). Fewer patients qualified for TNT (16%), and only 8% met the criteria for IMPROVE-IT and REDUCE-IT.

Common reasons for ineligibility across trials included lipid levels outside the permitted ranges, use of other LLT, absence of clinically evident ASCVD per trial definitions, and, in the case of CLEAR Outcomes, lack of statin intolerance (*Figure 1* and Supplementary material online, *Figures S1*  *and S2*).

### Differences in clinical characteristics between trial-eligible and trial-ineligible patients

Among CAD patients, those eligible for HPS were more often female and non-smokers, yet had a similar 10-year predicted absolute CV risk as ineligible patients (*Table 1*). TNT-eligible patients were younger, had lower SBP and LDL-C, and correspondingly had a lower 10-year predicted absolute CV risk (16 vs. 23%). FOURIER-eligible patients were more often current smokers and more likely to have CV comorbidities, with a concurrently higher 10-year predicted absolute CV risk (21 vs. 16%). CLEAR Outcomes required statin intolerance for inclusion, as reflected by significantly lower LLT use (12 vs. 94%) and higher LDL-C levels among eligible patients. This pattern was consistent in CeVD and PAD patients (see Supplementary material online, *Tables S2 and S3*).

**Table 1:** Clinical characteristics of trial-eligible and trial-ineligible UCC-SMART patients with coronary artery disease

	HPS *statin vs. placebo*		TNT *high vs. low dose statin*		IMPROVE-IT *ezetimibe*		FOURIER *PCSK9i*		REDUCE-IT *icosapent ethyl*		CLEAR Outcomes *bempedoic acid*	
	Ineligible *N = 830*	Eligible *N = 4843*	Ineligible *N = 1550*	Eligible *N = 4123*	Ineligible *N = 3771*	Eligible *N = 1902*	Ineligible *N = 3606*	Eligible *N = 2067*	Ineligible *N = 4855*	Eligible *N = 818*	Ineligible *N = 5183*	Eligible *N = 490*
**Patient characteristics**												
Age (years)	59 ± 12	62 ± 9^a^	64 ± 11	61 ± 9^a^	60 ± 10	64 ± 8^a^	61 ± 10	62 ± 9	62 ± 10	61 ± 8	62 ± 9	62 ± 10
Male sex	751 (90)	3825 (79)^a^	1259 (81)	3317 (80)	3050 (81)	1526 (80)	2911 (81)	1665 (81)	3919 (81)	657 (80)	4219 (81)	357 (73)^a^
Current smoker	229 (28)	981 (20)^a^	353 (23)	857 (21)	869 (23)	341 (18)^a^	606 (17)	604 (29)^a^	1025 (21)	185 (23)	1115 (22)	95 (19)
**Medical history**												
Cerebrovascular disease	74 (9)	420 (9)	178 (12)	316 (8)^a^	330 (9)	164 (9)	214 (6)	280 (14)^a^	419 (9)	75 (9)	451 (9)	43 (9)
Peripheral artery disease	72 (9)	269 (6)^a^	130 (8)	211 (5)^a^	243 (6)	98 (5)^a^	104 (3)	237 (11)^a^	294 (6)	47 (6)	295 (6)	46 (9)^a^
Abdominal aortic aneurysm	50 (6)	216 (4)	93 (6)	173 (4)^a^	194 (5)	72 (4)^a^	127 (4)	139 (7)^a^	229 (5)	37 (5)	234 (5)	32 (6)
Diabetes mellitus	176 (21)	912 (19)	302 (19)	786 (19)	661 (18)	427 (22)^a^	688 (19)	400 (19)	857 (18)	231 (28)^a^	1011 (19)	77 (16)^a^
10-year risk of recurrent CVD^b^ (%)	18 [13–28]	18 [12–25]	23 [16–33]	16 [12–23]^a^	17 [12–26]	18 [13–25]	16 [11–23]	21 [15–30]^a^	18 [12–26]	18 [13–25]	17 [12–25]	22 [16–31]^a^
**Medication**												
Blood pressure-lowering therapy	756 (91)	4397 (91)	1401 (90)	3752 (91)	3426 (91)	1727 (91)	3255 (90)	1898 (92)^a^	4393 (90)	760 (93)^a^	4752 (92)	401 (82)^a^
Glucose-lowering therapy	121 (15)	629 (13)	215 (14)	535 (13)	462 (12)	288 (15)^a^	475 (13)	275 (13)	586 (12)	164 (20)^a^	700 (14)	50 (10)^a^
Antithrombotic therapy	702 (85)	4364 (90)^a^	1321 (85)	3745 (91)^a^	3365 (89)	1701 (89)	3222 (89)	1844 (89)	4335 (89)	731 (89)	4684 (90)	382 (78)^a^
Lipid-lowering therapy	698 (84)	4230 (87)^a^	1353 (87)	3575 (87)	3273 (87)	1655 (87)	3144 (87)	1784 (86)	4170 (86)	758 (93)^a^	4871 (94)	57 (12)^a^
Statins	662 (80)	4167 (86)^a^	1308 (84)	3521 (85)	3194 (85)	1635 (86)	3076 (85)	1753 (85)	4075 (84)	754 (92)^a^	4829 (93)	0 (0)^a^
Ezetimibe	87 (10)	384 (8)^a^	144 (9)	327 (8)	408 (11)	63 (3)^a^	315 (9)	157 (8)	407 (8)	65 (8)	431 (8)	40 (8)
**Physical examination**												
Systolic blood pressure (mmHg)	137 ± 20	136 ± 20	142 ± 24	134 ± 17^a^	135 ± 20	137 ± 19^a^	137 ± 20	134 ± 18^a^	136 ± 20	135 ± 18^a^	136 ± 20	136 ± 17
Diastolic blood pressure (mmHg)	80 ± 11	80 ± 11	82 ± 13	79 ± 10^a^	80 ± 11	80 ± 11	81 ± 11	79 ± 10^a^	80 ± 11	79 ± 9^a^	80 ± 11	80 ± 10
Body mass index (kg/m²)	27 ± 4	27 ± 4	27 ± 4	27 ± 4	27 ± 4	27 ± 4	27 ± 4	28 ± 4^a^	27 ± 4	29 ± 4^a^	27 ± 4	27 ± 4
**Laboratory results**												
Total cholesterol (mmol/L)	5.5 ± 1.7^c^	5.7 ± 1.2^ac^	4.9 ± 1.3	4.2 ± 0.9^a^	4.6 ± 1.2	3.9 ± 0.6^a^	4.2 ± 1.1	4.7 ± 0.9^a^	4.4 ± 1.1	4.1 ± 0.5^a^	4.3 ± 1.0	5.7 ± 0.9^a^
HDL-C (mmol/L)	1.2 ± 0.3	1.2 ± 0.3	1.2 ± 0.3	1.2 ± 0.3^a^	1.2 ± 0.3	1.2 ± 0.3	1.2 ± 0.3	1.2 ± 0.3	1.2 ± 0.3	1.0 ± 0.2^a^	1.2 ± 0.3	1.2 ± 0.3
LDL-C (mmol/L)	2.4 ± 1.0	2.5 ± 0.9^a^	2.7 ± 1.1^d^	2.1 ± 0.6^ad^	2.7 ± 1.0	2.1 ± 0.4^a^	2.3 ± 0.9	2.7 ± 0.8^a^	2.5 ± 0.9	2.0 ± 0.4^a^	2.4 ± 0.8	3.7 ± 0.8^a^
Triglycerides (mmol/L)	1.4 [1.0–2.0]	1.3 [1.0–1.9]^a^	1.4 [1.0–2.0]	1.3 [1.0–1.9]^a^	1.4 [1.0–2.0]	1.2 [0.9–1.7]^a^	1.3 [0.9–1.8]	1.5 [1.1–2.0]^a^	1.2 [0.9–1.7]	2.0 [1.7–2.6]^a^	1.3 [1.0–1.9]	1.4 [1.1–2.0]^a^
HbA1c (%)	5.6 [5.4–6.0]	5.6 [5.4–6.0]	5.7 [5.4–6.0]	5.6 [5.4–6.0]	5.6 [5.4–6.0]	5.7 [5.4–6.1]^a^	5.6 [5.4–6.0]	5.7 [5.4–6.0]^a^	5.6 [5.4–6.0]	5.8 [5.5–6.3]^a^	5.6 [5.4–6.0]	5.6 [5.4–5.9]
eGFR (mL/min/1.73 m^2^)	79 ± 21	77 ± 16^a^	75 ± 19	79 ± 17^a^	79 ± 18	74 ± 16^a^	78 ± 17	76 ± 17^a^	78 ± 17	77 ± 17	78 ± 17	76 ± 16^a^

Data are presented as count (%) for categorical variables, median [interquartile range] for 10-year risk of recurrent CVD, triglycerides and HbA1c and mean ± standard deviation for other continuous variables.

UCC-SMART, Utrecht Cardiovascular Cohort–Second Manifestations of ARTerial disease; HPS, Heart Protection Study; TNT, Treating to New Targets trial; IMPROVE-IT, IMProved Reduction of Outcomes: Vytorin Efficacy Intervention Trial; FOURIER, Further Cardiovascular Outcomes Research with PCSK9 Inhibition in Subjects with Elevated Risk; PCSK9i, proprotein convertase subtilisin/kexin type 9 inhibitor; REDUCE-IT, Reduction of Cardiovascular Events with Icosapent Ethyl–Intervention Trial; CLEAR Outcomes, Cholesterol Lowering via Bempedoic Acid, an ACL-Inhibiting Regimen Outcomes trial; N, number; CVD, cardiovascular disease; HDL-C, high-density lipoprotein cholesterol; LDL-C, low-density lipoprotein cholesterol; HbA1c, glycated haemoglobin; eGFR, estimated glomerular filtration rate [calculated with Chronic Kidney Disease Epidemiology Collaboration (CKD-EPI) formula].

^a^Statistically significant difference between trial-eligible and ineligible patients (*P*-value <0.05).

^b^Calculated using the SMART2 risk score.^26^

^c^Recalculated to untreated levels.

^d^Recalculated to levels under atorvastatin 10 mg/day.

Among CeVD patients, SPARCL excluded individuals with coronary heart disease (CHD) and PAD. Consequently, SPARCL-eligible patients were less likely to have CV comorbidities and had a lower 10-year predicted absolute CV risk (16 vs. 20%) than ineligible patients (see Supplementary material online, *Table S2*). In contrast, patients eligible for TST, IMPROVE-IT, FOURIER, and REDUCE-IT were generally older, predominantly male, used more medications, and more often had CV comorbidities and lower estimated glomerular filtration rate than ineligible patients, resulting in a higher 10-year predicted absolute CV risk.

Among PAD patients, those eligible for HPS were largely comparable to ineligible patients, aside from some demographic differences (see Supplementary material online, *Table S3*). TNT and IMPROVE-IT required prior CHD or acute coronary syndrome (ACS) for inclusion, resulting in substantially higher 10-year predicted absolute CV risk among eligible compared with ineligible patients (29 vs. 21% for TNT and 29 vs. 22% for IMPROVE-IT). Like CAD and CeVD patients, REDUCE-IT-eligible PAD patients had more cardiometabolic comorbidities and higher triglyceride (TG) levels than their ineligible counterparts.

### Differences in risk of recurrent cardiovascular events and all-cause mortality between trial-eligible and trial-ineligible patients

**Figure 2:**
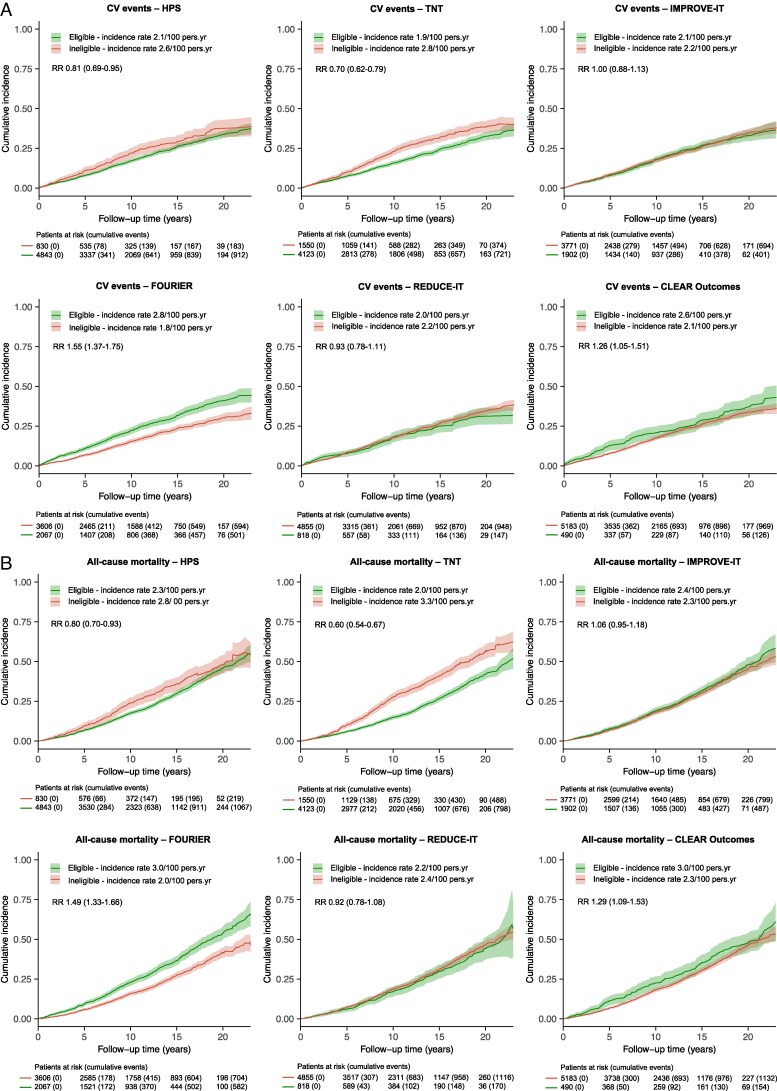
Cumulative incidence of recurrent CV events (*A*) and all-cause mortality (*B*) in trial-eligible and trial-ineligible UCC-SMART patients with coronary artery disease. Rate ratios are presented with 95% confidence intervals for trial-eligible patients compared with trial-ineligible patients. UCC-SMART, Utrecht Cardiovascular Cohort–Second Manifestations of ARTerial disease; CV, cardiovascular; pers.yr, person-years; RR, rate ratio; HPS, Heart Protection Study; TNT, Treating to New Targets trial; IMPROVE-IT, IMProved Reduction of Outcomes: Vytorin Efficacy Intervention Trial; FOURIER, Further Cardiovascular Outcomes Research with PCSK9 Inhibition in Subjects with Elevated Risk; PCSK9, proprotein convertase subtilisin/kexin type 9; REDUCE-IT, Reduction of Cardiovascular Events with Icosapent Ethyl–Intervention Trial; CLEAR Outcomes, Cholesterol Lowering via Bempedoic Acid, an ACL-Inhibiting Regimen Outcomes trial.

In CAD patients, cumulative incidences of recurrent CV events and all-cause mortality were comparable between eligible and ineligible groups across most trials (*Figure 2* and Supplementary material online, *Table S4*). However, FOURIER-eligible CAD patients had slightly higher cumulative incidences of both outcomes, consistent with their less favourable CV risk profile at baseline. Conversely, TNT-eligible CAD patients exhibited slightly lower cumulative incidences of recurrent CV events and all-cause mortality, which aligned with their more favourable baseline risk profile.

In CeVD patients, cumulative incidences of recurrent CV events and all-cause mortality were generally higher in trial-eligible patients than in ineligible patients, reflecting their higher baseline CV risk (see Supplementary material online, *Figure S3* and Supplementary material online, *Table S4*). The only exception was SPARCL, where eligible patients had a slightly better prognosis than ineligible patients, likely due to the trial's selection of CeVD patients without additional CV comorbidities.

In PAD patients, the prognosis of trial-eligible and ineligible patients was largely similar across most trials (see Supplementary material online, *Figure S4* and Supplementary material online, *Table S4*). However, for TNT and IMPROVE-IT, cumulative incidences of recurrent CV events and all-cause mortality were higher in eligible patients than in ineligible patients, which corresponded with their less favourable baseline CV risk profile.

### Sensitivity analysis

A sensitivity analysis restricted to patients enrolled from 2010 onward revealed comparable trial eligibility proportions to those observed in the full cohort, with minimal variation across most trials and ASCVD subtypes (see Supplementary material online, *Table S5*). Similarly, rate ratios for recurrent CV events and all-cause mortality for trial-eligible compared with ineligible patients showed consistent patterns between the full study period (2000–23) and the contemporary subset (2010–23) (see Supplementary material online, *Table S6*).

## Discussion

Eligibility for lipid-lowering RCTs varies substantially among patients with CAD, CeVD, and PAD, as well as across different trials, ranging from 7 to 85%. Although trial-eligible and ineligible patients exhibit differences in clinical characteristics, their long-term risks of recurrent CV events and all-cause mortality are largely comparable.

The observed variability in eligibility proportions may be attributed to a shift in trial design over time. Earlier trials, such as HPS and TNT, used broad inclusion criteria to establish the foundational efficacy and safety of statins. These trials primarily targeted patients with established CAD without imposing stringent lipid thresholds, reflecting the limited therapeutic options available at the time. In contrast, more recent trials, such as FOURIER, REDUCE-IT, and CLEAR Outcomes, implemented stricter inclusion criteria, focusing on subgroups with substantial residual CV risk or statin intolerance. This evolution in trial design aims to address unmet clinical needs, improving the clinical relevance of trial findings for high-risk populations. However, stricter eligibility criteria may also limit the representativeness of these trials for the broader ASCVD population.

In the present study, CAD patients were most frequently eligible for lipid-lowering trials, particularly for earlier trials such as HPS and TNT, consistent with their broad inclusion criteria. In contrast, CeVD and PAD patients were less frequently eligible, especially for trials such as TNT and IMPROVE-IT, which explicitly required a history of CHD or ACS for inclusion, thereby excluding many patients with CeVD and PAD. More recent trials, including REDUCE-IT and CLEAR Outcomes, had consistently low eligibility proportions across all ASCVD subtypes, reflecting their stringent criteria for TG and LDL-C levels and statin intolerance.

It is often believed that trial-eligible patients tend to have fewer risk factors and lower multimorbidity than ineligible patients, leading to a more favourable prognosis. However, while eligibility proportions and clinical characteristics varied across trials, our findings indicate that trial-eligible and ineligible patients generally had comparable risks of recurrent CV events and all-cause mortality. These comparable outcomes suggest that lipid-lowering trials do not systematically exclude patients with significantly different prognoses. This supports their broad representativeness of contemporary real-world ASCVD populations in terms of risk.

Notable differences in prognosis were observed for CeVD and PAD patients eligible for IMPROVE-IT, with rate ratios up to 2 for CV events and all-cause mortality, compared with a rate ratio of 1 for CAD patients. These differences likely stem from the trial’s selection of ACS patients. Since no dedicated RCTs of ezetimibe exist for CeVD and PAD populations, we applied the ACS-focused IMPROVE-IT criteria to these subgroups. Consequently, CeVD and PAD patients were only eligible if they had both their primary disease and a history of ACS. This resulted in a high-risk subgroup with a worse baseline prognosis than the broader CeVD and PAD populations, likely explaining the higher event rates observed in trial-eligible groups. These findings underscore the limitations of applying ACS-based eligibility criteria to non-ACS populations, rather than reflecting inherent prognostic differences between eligible and ineligible CeVD and PAD patients.

Similarly, CAD and CeVD patients eligible for FOURIER had higher risks of CV events and all-cause mortality, likely due to the trial’s focus on high-risk secondary prevention populations with elevated LDL-C levels despite statin therapy. In contrast, PAD patients eligible for FOURIER had similar risks of CV events and mortality as ineligible patients. This may be explained by the systemic and diffuse nature of atherosclerosis in PAD, which inherently places many individuals in the high-risk category.^27,28^ This is further supported by our finding that the proportion of PAD patients meeting FOURIER’s eligibility criteria was nearly twice as high as that of CeVD and CAD patients (65% compared with 31 and 36%, respectively).

The present study builds on prior research on the applicability of lipid-lowering trials to real-world patients. A systematic review and meta-analysis of 42 RCTs found that many lipid-lowering trials excluded individuals with common comorbidities, including advanced kidney disease, heart failure, cancer, and immune-related disorders, while also limiting enrolment of older adults and women.^11^ While the review identified low eligibility proportions for these subgroups, it was not assessed whether eligible and ineligible patients differed in clinical characteristics or prognosis. The applicability of REDUCE-IT was previously assessed in the REACH and CLARIFY registries, which reported eligibility proportions of 11% for ASCVD patients and 16% for stable CAD patients, respectively.^29,30^ These findings align with the results of the present study, where 8–14% of ASCVD patients met REDUCE-IT criteria. The REACH registry reported comparable incidence rates of CV events between REDUCE-IT-eligible and ineligible patients, consistent with our study.^30^ Two separate studies examined lipid-related inclusion criteria for REDUCE-IT and FOURIER in individuals with prior ASCVD.^31,32^ Both studies reported higher eligibility proportions than the present study, likely because they focused on lipid thresholds rather than applying all trial eligibility criteria. However, neither study compared clinical characteristics and outcomes between trial-eligible and ineligible real-world patients.

Our finding that trial-eligible and ineligible ASCVD patients had comparable outcomes aligns with previous studies on the applicability of sodium-glucose cotransporter 2 inhibitor trials and trials on antithrombotic treatment intensification in ASCVD populations.^33,34^ In contrast, in heart failure with reduced ejection fraction (HFrEF), ineligible patients have been shown to have worse outcomes than eligible ones.^35,36^ This discrepancy likely reflects differences in trial design. Lipid-lowering trials commonly use risk-enhancing inclusion criteria to selectively include high-risk patients, thereby increasing event rates and trial efficiency. In HFrEF trials, however, patients with poor prognosis are typically excluded due to concerns about tolerability or limited benefit. Given the inherently high risk of morbidity and mortality in HFrEF, additional enrichment is less necessary.^37,38^ Consequently, ineligibility in HFrEF is often a marker of clinical vulnerability, whereas in lipid-lowering trials, it may simply reflect the absence of enrichment criteria rather than a worse baseline risk.

The present study may have several implications for clinical practice. The findings reinforce the continued relevance of phase 3 lipid-lowering RCTs in informing secondary prevention guidelines. The overall similarity in prognosis between trial-eligible and ineligible patients implies that these trials do not selectively enrol only healthy individuals or those at very high CV risk. Thus, the results suggest that lipid-lowering trials remain broadly applicable to real-world ASCVD populations. However, over time, trial designs have become more selective, increasingly focusing on high-risk subgroups with unmet clinical needs. While this shift improves therapeutic precision, it reduces generalizability. This paradox is particularly relevant to clinical decision-making: newer trials, such as REDUCE-IT and CLEAR Outcomes, often have restrictive eligibility criteria, thereby excluding a substantial proportion of real-world patients. Clinicians should therefore consider whether individual patients align with trial populations when prescribing therapies such as icosapent ethyl and bempedoic acid. For subgroups such as CeVD and PAD, where dedicated trials remain scarce, treatment recommendations frequently rely on extrapolation from CHD- or ACS-focused studies. To improve the inclusivity and precision of future guideline recommendations, more research in CeVD and PAD populations is needed. This could be achieved through well-designed real-world evidence studies, subgroup analyses in future trials, or broader inclusion criteria that better represent these patient groups.

The present study has several strengths, which include the systematic evaluation and direct comparison of guideline-informing lipid-lowering trials across three distinct ASCVD subtypes, and the large, well-characterized cohort with comprehensive baseline and long-term outcome data. The cohort reflects contemporary real-world clinical practice, as patients with CAD, CeVD, and PAD were recruited from a broad catchment area in the Netherlands. By rigorously applying eligibility criteria and analysing outcomes across multiple lipid-lowering agents recommended in clinical guidelines, our findings provide insight into the representativeness of these trials across different therapeutic strategies. Additionally, we examined how eligibility proportions and patient outcomes varied across trials conducted in different time periods, with a sensitivity analysis from 2010 onward demonstrating consistent patterns. This reinforces the reliability of our findings despite evolving patient characteristics and treatment strategies over time.

Limitations of this study should be acknowledged. While the UCC-SMART cohort provides detailed data on a broad population of ASCVD patients, it is a single-centre study conducted in the Netherlands—a low-risk region—which may limit generalizability to higher-risk populations or countries with different healthcare systems. In addition, certain trial exclusion criteria, such as hepatic dysfunction, inflammatory muscle disease, nephrotic syndrome, and severe heart failure, could not be applied due to unavailable data. However, as these conditions are relatively uncommon, their omission likely had minimal impact on eligibility estimates. For pragmatic reasons, we selected one representative trial per lipid-lowering agent, prioritizing size and scope. However, this approach is inherently arbitrary, and trials with different inclusion and exclusion criteria may yield different results. To align with trial protocols, lipid levels were recalculated to untreated total cholesterol (HPS), untreated LDL-C (SPARCL), and LDL-C under atorvastatin 10 mg (TNT). While necessary, inter-individual variability in the lipid-lowering effect of medication could not be accounted for. Lastly, the UCC-SMART cohort included stable ASCVD patients, whereas IMPROVE-IT enrolled patients with recent ACS, which may affect comparability.

In conclusion, the eligibility of real-world ASCVD patients for lipid-lowering RCTs varied substantially across trials and ASCVD subtypes. Despite differences in clinical characteristics, trial-eligible and ineligible patients generally had comparable risks of recurrent CV events and all-cause mortality. These findings suggest that guideline-informing lipid-lowering trials are broadly representative of contemporary real-world ASCVD patients.

## Supplementary Material

qcaf091_Supplementary_Data

## Data Availability

De-identified individual participant data, along with the corresponding data dictionary, are available to researchers upon reasonable request and following approval by the UCC-SMART study group. Data access requests can be submitted via email to ucc-smart@umcutrecht.nl.
