# Supplementary material for: Applicability of guideline-informing lipid-lowering clinical trials to contemporary real-world patients with atherosclerotic cardiovascular disease
Source: Eur Heart J Qual Care Clin Outcomes. 2025 Aug 29;11(8):1359–68. doi: 10.1093/ehjqcco/qcaf091 (PMC12714395; doi:10.1093/ehjqcco/qcaf091)
Supplement: qcaf091_Supplementary_Data [file qcaf091_supplementary_data.pdf]

Applicability of guideline-informing lipid-lowering clinical trials to contemporary real-world patients with atherosclerotic cardiovascular disease

Supplementary Material

Supplementary Table 1. Trial eligibility criteria and their operationalization within the UCC-SMART study.

HPS[14]

**Inclusion criteria**	**Operationalization in UCC-SMART**
Male or female aged about 40 to 80 years	Age at inclusion ≥40 and <80 years
High risk of CHD death over the next 5 years:	
- Coronary disease: definite or probable clinical diagnosis of MI, unstable angina, stable angina, PTCA or CABG	- MI, PCI, or CABG ever<br>Inclusion because of MI, angina pectoris with proven stenosis on coronary angiogram, ≥1 vessel disease on coronary angiography, or coronary syndrome requiring PCI or CABG
- Occlusive disease of non-coronary arteries: clinical, angiographic or ultrasound diagnosis of carotid artery stenosis (e.g. TIA or non-disabling stroke not thought to be hemorrhagic), carotid endarterectomy, leg artery stenosis (e.g. intermittent claudication) or surgery	- Ischemic stroke ever<br>Inclusion because of duplex ultrasound confirmed asymptomatic carotid artery stenosis, TIA, cerebral infarction, or ischemic retinal syndrome<br>Previous carotid revascularization, duplex ultrasound confirmed carotid artery stenosis at baseline<br>Inclusion because of Fontaine classification ≥II (confirmed by ABI ≤0.90 at rest and/or ≥20% post-exercise decrease)<br>History of lower extremity bypass surgery, percutaneous transluminal angioplasty revascularization, or amputation for arterial vascular disease
- DM (whether type 1 or type 2) clinical diagnosis of insulin-dependent or non-insulin-dependent DM	- Inclusion because of DM, DM in medical history
- Treated hypertension: use of antihypertensive drug therapy in male aged ≥65 years	- Male sex and age at inclusion ≥65 years and use of antihypertensive medication at baseline
No clear indications for the study treatments: the patient is not already taking HMG-CoA reductase inhibitors or high-dose vitamin E supplements, and neither the patient nor the patient's doctor considers there to be any definite need to do so	*Criterion regarding HMG-CoA reductase inhibitors omitted because statins are now standard care for patients with established ASCVD; information on vitamin E supplements not available*

**Exclusion criteria**	
No clear contraindications to the study treatments:	
- Baseline plasma cholesterol <3.5 mmol/L	- Baseline total cholesterol <3.5 mmol/L; for participants using statins at baseline, untreated values estimated by adjusting measured total cholesterol levels to account for ~26% reduction due to statin therapy
- Chronic liver disease (i.e. cirrhosis or hepatitis) or abnormal liver function (i.e. ALT >1.5x ULN)	- *Information not available*
- Severe renal disease or evidence of renal impairment (i.e. creatinine >2x ULN)	- Baseline creatinine >208 µmol/L for men and >180 µmol/L for women; *ULN according to local laboratory cutoff values for men and women*
- Inflammatory muscle disease (such as dermatomyositis or polymyositis) or CK >3x ULN	- *Information not available*

- Concurrent treatment with cyclosporin (or a condition likely to result in organ transplantation and the need for cyclosporin)	- Use of cyclosporin at baseline; *other information not available*
- Concurrent treatment with fibrates or high-dose niacin. (N.B. Patients on cholesterol-lowering diets or drugs—other than HMG-CoA reductase inhibitors, fibrates or high-dose niacin—could still be entered in the study)	- Use of fibrates or niacin at baseline
- Child-bearing potential (i.e. pre-menopausal woman who is not sterilized or using a reliable method of contraception)	- *Information not available*
No other predominant medical problem:	
- Severe heart failure or some importantly life-threatening condition other than vascular disease (such as very severe chronic airways disease or any cancer other than non-melanoma skin cancer)	- Short life expectancy is exclusion criterion
- Psychiatric disorder, senility or physical disability (such as severely disabling stroke)	- Independent in most daily activities is inclusion criterion (Rankin scale ≤3); *other information not available*
- Recent history of alcohol or drug abuse	- Self-reported weekly alcohol intake >21 standard drinks; *information on drug abuse not available*

TNT[16]

Inclusion criteria

Men and women aged 35 to 75 years	Age at inclusion ≥35 and <75 years
Clinically evident CHD, defined as previous MI, previous or present angina with objective evidence of atherosclerotic CHD, and who have undergone a coronary revascularization procedure	MI, PCI, or CABG ever<br>Inclusion because of MI, angina pectoris with proven stenosis on coronary angiogram, ≥1 vessel disease on coronary angiography, or coronary syndrome requiring PCI or CABG

Exclusion criteria

Mean LDL-C ≥3.4 mmol/L with atorvastatin 10 mg daily at the end of the run-in period	Baseline LDL-C ≥3.4 mmol/L; for participants not using LLT, baseline LDL-C recalculated to atorvastatin 10 mg equivalent by applying 37% reduction to untreated LDL-C values
Hypersensitivity to statins	*Information not available*
Active liver disease or hepatic dysfunction defined as ALT or AST >1.5x ULN	*Information not available*
Women who are pregnant or breastfeeding	Pregnancy is exclusion criterion; *information on breastfeeding not available*
Patients with nephrotic syndrome	*Information not available*
Uncontrolled DM (as defined by the investigator)	Baseline HbA1c >10%; *aligned with cutoff values from other trials*
Uncontrolled hypothyroidism (as defined by the investigator)	Baseline TSH >7.5 mIU/L; *aligned with cutoff values from other trials*
Uncontrolled hypertension (as defined by the investigator)	Baseline SBP >180 mmHg; *aligned with cutoff values from other trials*

A MI, coronary revascularization procedure or severe/unstable angina within 1 month of screening	*Assumption nobody since inclusion is generally > 1 month after acute event*
Any planned surgical procedure for the treatment of atherosclerosis	*Information not available*
An ejection fraction <30%	*Information not available*
Hemodynamically important valvular disease	*Information not available*
Gastrointestinal disease limiting drug absorption or partial ileal bypass	*Information not available*
Any nonskin malignancy, malignant melanoma or other survival-limiting disease	Short life expectancy is exclusion criterion; *information on malignancy at baseline not available*
Unexplained CK levels >6x ULN	*Information not available*
Concurrent therapy with long-term immunosuppressants	*Information not available*
Concurrent therapy with lipid-regulating drugs not specified as study treatment in the protocol	*Criterion omitted because LLT is now standard care for patients with established ASCVD*
History of alcohol abuse	Self-reported weekly alcohol intake >21 standard drinks
Participation in another clinical trial concurrently or <30 days before screening	*Information not available*

IMPROVE-IT[18]

Inclusion criteria

Men and women aged at least 50 years	Age at inclusion ≥50 years
Subjects with planned PCIs as management for the qualifying ACS event were to undergo a PCI prior to randomization and within the 10-day period after the initial hospitalization for the event	*Information not available*
Hospitalized within the preceding 10 days for an ACS (an acute MI, with or without ST segment elevation on ECG, **or**	MI, PCI, or CABG ever<br>Inclusion because of MI or coronary syndrome requiring PCI or CABG
High-risk unstable angina, defined as symptoms of cardiac ischemia and any 1 of the following criteria:<br>a) ECG changes by either of the following:<br>[1] New or presumably new ST-segment depression ≥0.1 mV in at least 2 contiguous ECG leads; or<br>[2] Transient (<30 minutes) ST-segment elevation ≥0.1 mV in at least 2 contiguous ECG leads.<br>b) Any of the following cardiovascular biomarkers elevated >ULN:	Inclusion because of angina pectoris **and**:<br>- *Information not available*<br>- *Information not available*

[3] Troponin I;<br>[4] Troponin T; and/or<br>[5] Creatine kinase-MB fraction	
c) DM;	- Inclusion because of DM, DM in medical history
d) History of prior MI;	- MI ever, inclusion because of MI
e) History of PAD;	- Fontaine classification ≥II, history of lower extremity bypass surgery, percutaneous transluminal angioplasty revascularization, or amputation for arterial vascular disease
f) History of CeVD;	- TIA, cerebral infarction, ischemic retinal syndrome, carotid surgery, or angioplasty in medical history
g) History of CABG ≥3 years prior to entry	- CABG ever
h) Multivessel CAD previously documented by catheterization (2 or 3 vessels with ≥50% stenosis) including the catheterization performed during the index admission for the qualifying event	- Inclusion because of ≥2 vessel disease on coronary angiography
LDL-C concentration ≥1.3 mmol/L and ≤3.2 mmol/L for lipid-therapy naïve subjects	Baseline LDL-C ≥1.3 mmol/L and ≤3.2 mmol/L and no use of LLT at baseline
LDL-C concentration ≥1.3 mmol/L and ≤2.6 mmol/L for subjects receiving chronic prescription LLT (receiving any LLT continuously for >4 weeks prior to and continuing until the qualifying ACS hospital admission)	Baseline LDL-C ≥1.3 mmol/L and ≤2.6 mmol/L and use of LLT at baseline
Plasma TG level ≤4.0 mmol/L	Baseline TG ≤4.0 mmol/L
Clinical laboratory tests within reference ranges or clinically acceptable to the investigator/sponsor	*Information not available*
Use of a medically accepted method of contraception while receiving protocol-specified medication and for 6 weeks after stopping the medication for women of child-bearing potential, or agreement to do so if not currently sexually active but becoming sexually active while participating in the study	*Information not available*

Exclusion criteria

Subject was clinically unstable, i.e. any of the following events <24 hours prior to screening/randomization:<br>a) Hemodynamic events:<br>1) Hypotension, defined as sustained SBP of <90 mmHg due to cardiac failure with associated symptoms;<br>2) Unstable or severe pulmonary edema/decompensated congestive heart failure;<br>3) Acute mitral regurgitation;<br>4) Acute ventricular septal defect.<br>b) Recurrent symptoms of cardiac ischemia:<br>c) Stroke or TIA;<br>d) Arrhythmic events:<br>1) Ventricular fibrillation;	*Assumption nobody since inclusion is generally >1 month after acute event*

2) Sustained ventricular tachycardia lasting >30 seconds or in association with symptoms;<br>3) Complete heart block;<br>4) High grade second degree heart block	
Subjects who planned or underwent CABG in response to the initial episode of ACS	*Information not available*
Concomitant therapy with any of the following medications: cyclosporine, diltiazem, danazol, amiodarone, verapamil, niacin, fibrates as concomitant medications or any of the potent CYP3A4 inhibitors, itraconazole, ketoconazole, erythromycin, clarithromycin, and telithromycin, HIV protease inhibitors, nefazodone, probucol, resins, grapefruit juice >1 quart/day, torceptrapib, and any investigational drugs. Routes of administration other than oral or parenteral (eg, topical, intraocular, otic) of antifungal or antibiotics were acceptable.	Use of cyclosporine, niacin, fibrates, oral or parenteral itraconazole or ketoconazole, or HIV protease inhibitors at baseline; *other information not available*
The investigator felt that discontinuation of existing lipid-lowering regimen posed a risk to the subject	*Information not available*
Use of chronic LLT with LDL-C lowering potency greater than simvastatin 40 mg†	Use of LLT with LDL-C lowering potency greater than simvastatin 40 mg at baseline†
Allergy/sensitivity to any statin, ezetimibe, and/or their excipients	*Information not available*
Active liver disease or persistent serum transaminase elevations (≥2x ULN)	*Information not available*
Calculated creatinine clearance (Cockcroft-Gault) <30 mL/min or dialysis within 30 days	Cockcroft-Gault creatinine clearance <30 mL/min; *information on dialysis not available*
History of alcohol and/or drug abuse	Self-reported weekly alcohol intake >21 standard drinks; *information on drug abuse not available*
Pregnant or lactating women, or women intending to become pregnant	Pregnancy is exclusion criterion; *other information not available*
Any clinically significant condition or situation, other than the condition being studied that, in the opinion of the investigator, would interfere with the study evaluations or optimal participation in the study	*Information not available*
Use of any investigational drugs <30 days of screening/randomization	*Information not available*
Participation in any other clinical study involving an investigational drug or device with the following exceptions:<br>a) A subject participating in the EARLY-ACS Study was not necessarily excluded<br>b) A subject participating in clinical research of approved therapy being administered according to the therapy's labelled use was not to be excluded.	*Information not available*
Prior enrolment in this current study	*Information not available*

Subject who was part of the staff personnel directly involved with this study, or a family member of the investigational study staff	*Information not available*

FOURIER[19]

Inclusion criteria

Age ≥40 and ≤85 years	Age ≥40 and ≤85 years at inclusion
History of clinically evident CVD as evidenced by any of the following:	
- Diagnosis of MI	- MI ever, inclusion because of MI
- Diagnosis of non-hemorrhagic stroke (TIA does not qualify as stroke for inclusion)	- Ischemic stroke ever, inclusion because of cerebral infarction
- Symptomatic PAD, as evidenced by intermittent claudication with ABI <0.85, or peripheral arterial revascularization procedure, or amputation due to atherosclerotic disease	- Inclusion because of Fontaine classification ≥II (confirmed by ABI ≤0.90 at rest and/or ≥20% post-exercise decrease), history of lower extremity bypass surgery, percutaneous transluminal angioplasty revascularization, or amputation for arterial vascular disease
At least 1 major risk factor or at least 2 minor risk factors below:	
<u>Major risk factors (1 required):</u>	
- DM (type 1 or type 2)	- Inclusion because of DM, DM in medical history (type 1 or type 2)
- Age ≥65 years at randomization (and ≤85 years at time of informed consent)	- Age ≥65 years and ≤85 years at inclusion
- MI or non-hemorrhagic stroke within 6 months of screening	- MI ever, inclusion because of MI, ischemic stroke ever, inclusion because of cerebral infarction
- Additional diagnosis of MI or non-hemorrhagic stroke excluding qualifying MI or non-hemorrhagic stroke	- ≥1 of the following: MI ever, inclusion because of MI, ischemic stroke ever, inclusion because of cerebral infarction
- Current daily cigarette smoking	- Self-reported current smoking
- History of symptomatic PAD (intermittent claudication with ABI <0.85, or peripheral arterial revascularization procedure, or amputation due to atherosclerotic disease) if eligible by MI or stroke history	- Inclusion because of Fontaine classification ≥II (confirmed by ABI ≤0.90 at rest and/or ≥20% post-exercise decrease), history of lower extremity bypass surgery, percutaneous transluminal angioplasty revascularization, or amputation for arterial vascular disease **and** MI ever, inclusion because of MI, ischemic stroke ever, or inclusion because of cerebral infarction,
<u>Minor risk factors (2 required):</u>	
- History of non-MI related coronary revascularization	- Inclusion because of CABG or PCI, CABG or PCI ever
- Residual CAD with ≥40% stenosis in ≥2 large vessels	- *Information not available*
- Most recent HDL-C <1.0 mmol/L for men and <1.3 mmol/L for women by central laboratory before randomization	- Baseline HDL-C <1.0 mmol/L for men and <1.3 mmol/L for women
- Most recent hsCRP >2.0 mg/L by central laboratory before randomization	- Baseline hsCRP >2.0 mg/L
- Most recent LDL-C ≥3.4 mmol/L or non-HDL-C ≥4.1 mmol/L by central laboratory before randomization	- Baseline LDL-C ≥3.4 mmol/L or non-HDL-C ≥4.1 mmol/L
- Metabolic syndrome, defined as defined as ≥3 of the following:	- ≥3 of the following:
• waist circumference >102 cm (>40 in.) for men and >88 cm (>35 in.) for women (Asian men, including Japanese >90 cm; Asian women, except Japanese >80 cm; Japanese women >90 cm)	• baseline waist circumference >102 cm for men and >88 cm for women; *information on ethnicity not available*
• TG ≥150 mg/dL (1.7 mmol/L) by central laboratory at final screening	• baseline TG ≥1.7 mmol/L

• HDL-C <40 mg/dL (1.0 mmol/L) for men and <50 mg/dL (1.3 mmol/L) for women by central laboratory at final screening<br>• SBP ≥130 mmHg or DBP ≥85 mmHg or hypertension treated with medication<br>• fasting glucose ≥100 mg/dL (≥5.6 mmol/L) by central laboratory at final screening	• baseline HDL-C <1.0 mmol/L for men and <1.3 mmol/L for women<br>• baseline SBP ≥130 mmHg or DBP ≥85 mmHg or use of antihypertensive medication at baseline<br>• baseline glucose ≥5.6 mmol/L
Most recent fasting LDL-C ≥70 mg/dL (≥1.8 mmol/L) or non-HDL-C ≥ 100 mg/dL (≥2.6 mmol/L) by central laboratory during screening after ≥2 weeks of stable LLT	Baseline LDL-C ≥1.8 mmol/L or non-HDL-C ≥2.6 mmol/L
Most recent fasting TG ≤400 mg/dL (4.5 mmol/L) by central laboratory before randomization	Baseline TG ≤4.5 mmol/L
**Exclusion criteria**	
Subject must not be randomized within 4 weeks of their most recent MI or stroke	*Assumption nobody since inclusion is generally >1 month after acute event*
NYHA class III or IV, or last known left ventricular ejection fraction <30%	*Information not available*
Known hemorrhagic stroke at any time	Inclusion because of hemorrhagic stroke or subarachnoid hemorrhage; *information on hemorrhagic stroke in medical history not available*
Uncontrolled or recurrent ventricular tachycardia	*Information not available*
Planned or expected cardiac surgery or revascularization <3 months after randomization	*Information not available*
Uncontrolled hypertension defined as sitting SBP >180 mmHg or DBP >110 mmHg	Baseline SBP >180 mmHg or DBP >110 mmHg
Use of cholesteryl ester transfer protein inhibition treatment, mipomersen, or lomitapide <12 months prior to randomization. Fenofibrate therapy must be stable for ≥6 weeks prior to final screening at a dose that is appropriate for the duration of the study in the judgment of the investigator. Other fibrate therapy (and derivatives) are prohibited	Use of mipomersen, lomitapide, or fibrates at baseline; *fenofibrate not used by any of the participants so criterion regarding stable therapy was omitted, information on use of cholesteryl ester transfer protein inhibition treatment not available*
Prior use of PCSK9 inhibition treatment other than evolocumab or use of evolocumab <12 weeks prior to final lipid screening	Use of PCSK9 inhibitor at baseline
Untreated or inadequately treated hyperthyroidism or hypothyroidism as defined by TSH <LLN or >1.5x ULN, respectively, and free thyroxine levels that are outside normal range at final screening	Baseline TSH <0.35 mIU/L or >7.5 mIU/L (LLN and ULN according to local laboratory cutoff values); *information on free thyroxine levels not available*
Severe renal dysfunction, defined as an eGFR <20 mL/min/1.73m^2 at final screening	Baseline eGFR <20 mL/min/1.73m^2
Active liver disease or hepatic dysfunction, defined as AST or ALT >3x ULN as determined by central laboratory analysis at final screening	*Information not available*

Recipient of any major organ transplant (eg, lung, liver, heart, bone marrow, renal)	*Information not available*
Personal or family history of hereditary muscular disorders	*Information not available*
LDL or plasma apheresis <12 months prior to randomization	*Information not available*
Severe, concomitant non-cardiovascular disease that is expected to reduce life expectancy to <3 years	Short life expectancy is exclusion criterion
CK >5x ULN at final screening	*Information not available*
Known major active infection or major hematologic, renal, metabolic, gastrointestinal or endocrine dysfunction in the judgment of the investigator	*Information not available*
Malignancy (except non-melanoma skin cancers, cervical in-situ carcinoma, breast ductal carcinoma in situ, or stage 1 prostate carcinoma) within the last 10 years	*Information not available*
Subject has received drugs via a systemic route that have known major interactions with background statin therapy <1 month prior to randomization or is likely to require such treatment during the study period	*Information not available*
Currently enrolled in another investigational device or drug study, or <30 days since ending another investigational device or drug study(s), or receiving other investigational agent(s)	*Information not available*
Female subject who has either (1) not used acceptable method(s) of birth control for at least 1 month prior to screening or (2) is not willing to use such a method during treatment with investigational product and for an additional 15 weeks after the end of treatment with investigational product, unless the subject is sterilized or postmenopausal	*Information not available*
Subject is pregnant or breast feeding, or planning to become pregnant or to breastfeed during treatment with investigational product and/or <15 weeks after the end of treatment with investigational product	Pregnancy is exclusion criterion; *other information not available*
Known sensitivity to any of the active substances or their excipients to be administered during dosing	*Information not available*
Subject likely to not be available to complete all protocol-required study visits or procedures, to the best of the subject's and investigator's knowledge	*Information not available*
History or evidence of any other clinically significant disorder, condition or disease other than those outlined above that, in the opinion of the Investigator or Amgen physician, if consulted, may compromise the ability of the subject to give written informed consent,	*Information not available*

would pose a risk to subject safety, or interfere with the study evaluation, procedures or completion

REDUCE-IT[20]

Inclusion criteria

Men and women age ≥45 years and ≥1 of the following:	Age ≥45 years at inclusion and ≥1 of the following:
- Documented CAD (≥1 of the following primary criteria must be satisfied):	
a) Documented multivessel CAD (≥50% stenosis in ≥2 major epicardial coronary arteries, with or without antecedent revascularization)	- Inclusion because of ≥2 vessel disease on coronary angiography
b) Documented prior MI	- MI ever, inclusion because of MI
c) Hospitalization for high-risk non-ST-segment elevation ACS (with objective evidence of ischemia: ST-segment deviation or biomarker positivity)	- Inclusion because of CABG or PCI, CABG or PCI ever
- Documented cerebrovascular or carotid disease (1 of the following primary criteria must be satisfied):	
a) Documented prior ischemic stroke	- Inclusion because of cerebral infarction, ischemic stroke ever
b) Symptomatic carotid artery disease with ≥50% carotid arterial stenosis	- Duplex ultrasound confirmed stenosis or occlusion of ≥1 carotid artery with diameter reduction ≥50% and medical history of CeVD
c) Asymptomatic carotid artery disease with ≥70% carotid arterial stenosis per angiography or duplex ultrasound	- Duplex ultrasound confirmed stenosis or occlusion of ≥1 carotid artery with diameter reduction ≥70% and no medical history of CeVD
d) History of carotid revascularization (catheter-based or surgical)	- Carotid surgery or angioplasty in medical history
- Documented PAD (≥1 of the following primary criteria must be satisfied):	
a) ABI <0.9 with symptoms of intermittent claudication	- Inclusion because of Fontaine classification ≥II (confirmed by ABI ≤0.90 at rest and/or ≥20% post-exercise decrease)
b) History of aortoiliac or peripheral arterial intervention (catheter-based or surgical)	- History of lower extremity bypass surgery, percutaneous transluminal angioplasty revascularization, or amputation for arterial vascular disease, or history of aortoiliac intervention
**or**	
Men and women age ≥50 years with DM (type 1 or type 2) requiring treatment with medication and ≥1 of the following additional risk factors for CVD:	Age ≥50 years at inclusion and inclusion because of DM or DM in medical history (type 1 or type 2) and use of insulin or glucose-lowering therapy and ≥1 of the following:
- Men ≥55 years of age and women ≥65 years of age	- Age ≥55 years at inclusion for men or age ≥65 years at inclusion for women
- Cigarette smoker or stopped smoking <3 months before Visit 1	- Self-reported current smoking
- Hypertension (SBP ≥140 mmHg or DBP ≥90 mmHg) or on antihypertensive medication	- Baseline SBP ≥140 mmHg or DBP ≥90 mmHg or use of antihypertensive medication at baseline
- HDL-C ≤40 mg/dL for men or ≤50 mg/dL for women	- Baseline HDL-C ≤1.03 mmol/L for men or ≤1.30 mmol/L for women
- hsCRP >3.00 mg/L	- Baseline hsCRP >3.00 mg/L
- Renal dysfunction: creatinine clearance >30 and <60 mL/min	- Baseline eGFR >30 and <60 mL/min/m^2
- Retinopathy, defined as any of the following: nonproliferative retinopathy, preproliferative retinopathy, proliferative retinopathy, maculopathy, advanced diabetic eye disease, or a history of photocoagulation	- *Information not available*

- Micro- or macroalbuminuria. Microalbuminuria is defined as either a positive micral or other strip test, an albumin/creatinine ratio ≥2.5 mg/mmol, or an albumin excretion rate on timed collection ≥20 mg/min all on ≥2 successive occasions. Macroalbuminuria is defined as Albustix or other dipstick evidence of gross proteinuria, an albumin/creatinine ≥25 mg/mmol, or an albumin excretion rate on timed collection ≥200 mg/min all on ≥2 successive occasions	- Baseline albumin/creatinine ratio ≥2.5 mg/mmol; *other information not available*<br>Baseline albumin/creatinine ratio ≥25 mg/mmol; *other information not available*
- ABI <0.9 without symptoms of intermittent claudication (patients with ABI <0.9 with symptoms of intermittent claudication are counted under CV Risk Stratum 1)	- ABI <0.9 and no history of Fontaine classification ≥II
Fasting TG levels ≥150 mg/dL and <500 mg/dL§	Baseline TG ≥1.52 mmol/L and <5.63 mmol/L§
LDL-C >40 mg/dL and ≤100 mg/dL and on stable statin therapy (± ezetimibe) for ≥4 weeks prior to the LDL-C and TG qualifying measurements for randomization	Baseline LDL-C >1.06 mmol/L and <2.59 mmol/L
Women who are not pregnant, not breastfeeding, not planning on becoming pregnant, and using an acceptable form of birth control during the study (if of child-bearing potential)	Pregnancy is exclusion criterion; *other information not available*
Able to provide informed consent and adhere to study schedules	*Information not available*
Agree to follow and maintain a physician-recommended diet during the study	*Information not available*
**Exclusion criteria**	
Severe (NYHA class IV) heart failure	*Information not available*
Any life-threatening disease expected to result in death <2 years (other than CVD)	Short life expectancy is exclusion criterion
Diagnosis or laboratory evidence of active severe liver disease	*Information not available*
HbA1c >10.0% at screening	Baseline HbA1c >10.0%
Poorly controlled hypertension: SBP ≥200 mmHg or DBP ≥100 mmHg (despite antihypertensive therapy)	Baseline SBP ≥200 mmHg or DBP ≥100 mmHg and use of antihypertensive medication at baseline
Planned coronary intervention or any noncardiac major surgical procedure	*Information not available*
Known familial lipoprotein lipase deficiency (Fredrickson type I), apolipoprotein C-II deficiency, or familial dysbetalipoproteinemia (Fredrickson type III)	*Information not available*
Participation in another clinical trial involving an investigational agent <90 days prior to screening	*Information not available*
Intolerance or hypersensitivity to statin therapy	*Information not available*

Known hypersensitivity to fish and/or shellfish, or ingredients of the study product or placebo	*Information not available*
History of acute or chronic pancreatitis	*Information not available*
Malabsorption syndrome and/or chronic diarrhea	*Information not available*
Use of non–study-drug-related, non-statin lipid-altering medications, dietary supplements, or foods during the screening period (after Visit 1) and/or plans for use during the treatment/follow-up period, including:	
- Niacin (>200 mg/d) or fibrates (unless ≥28-day washout)	- Use of niacin or fibrates at baseline
- Any omega-3 fatty acid medications (unless ≥28-day washout)	- Use of omega-3 fatty acid medications at baseline
- Dietary supplements containing omega-3 fatty acids (eg, flaxseed, fish, krill, or algal oils; unless ≥28-day washout)	- *Information not available*
- Bile acid sequestrants (unless ≥7-day washout)	- Use of bile acid sequestrants at baseline
- PCSK9 inhibitors (unless ≥90-day washout)	- Use of PCSK9 inhibitors at baseline
Other medications (not indicated for lipid alteration):	
- Tamoxifen, estrogens, progestins, thyroid hormone therapy, systemic corticosteroids (local, topical, inhalation, or nasal corticosteroids are allowed), HIV-protease inhibitors that have not been stable for ≥28 days prior to the qualifying lipid measurements (TG and LDL-C) during screening	- Use of tamoxifen, estrogens, progestins, thyroid hormone therapy, systemic corticosteroids, or HIV-protease inhibitors at baseline
- Cyclophosphamide or systemic retinoids during the screening period (unless ≥28-day washout) and/or plans for use during the treatment/follow-up period	- Use of cyclophosphamide or systemic retinoids at baseline
Known AIDS (HIV-positive patients without AIDS are allowed)	*Information not available*
Requirement for peritoneal dialysis or hemodialysis for renal insufficiency or creatinine clearance <30 mL/min	Cockcroft-Gault creatinine clearance <30 mL/min; *other information not available*
Unexplained elevated CK concentration >5x ULN or elevation due to known muscle disease	*Information not available*
Any condition or therapy which, in the opinion of the investigator, might pose a risk to the patient or make participation in the study not in the patient's best interest	*Information not available*
Drug or alcohol abuse within the past 6 months, and inability/unwillingness to abstain from drug abuse and excessive alcohol consumption during the study	Self-reported weekly alcohol intake >21 standard drinks; *other information not available*
Mental/psychological impairment or any other reason to expect patient difficulty in complying with the requirements of the study or understanding the goal and potential risks of participating in the study	*Information not available*

CLEAR Outcomes[21]

Inclusion criteria

Age ≥18 years (or legal age of majority based on regional law, whichever is greater) and ≤85 years	Age ≥18 years and ≤85 years at inclusion
Patient-reported statin intolerance due to an adverse safety effect that started or increased during statin therapy and resolved or improved when statin therapy was discontinued resulting in an inability to tolerate:<br>• 2 or more statins at any dose, or<br>• 1 statin at any dose and unwilling to attempt a second statin or advised by a physician to not attempt a second statin<br>Please note that patients currently tolerating very low dose statin therapy (an average daily dose of rosuvastatin <5 mg, atorvastatin <10 mg, simvastatin <10 mg, lovastatin <20 mg, pravastatin <40 mg, fluvastatin <40 mg, or pitavastatin <2 mg) are considered to be intolerant to that low dose statin. Patients may continue taking very low dose statin therapy throughout the study provided that it is stable (used for at least 4 weeks prior to screening) and well tolerated.	*Assumption everybody using statin therapy at baseline was not statin intolerant*
Written confirmation by both patient and investigator that the patient is statin intolerant as defined above, aware of the benefit of statin use to reduce the risk of major adverse cardiovascular events including death, and also aware that many other patients who are unable to tolerate a statin are able to tolerate a different statin or dose	*Information not available*
Men and nonpregnant, nonlactating women. Women must be one of the following:<br>• Naturally postmenopausal defined as ≥1 year without menses and:<br>o ≥55 years, **or**<br>o <55 years with follicle-stimulating hormone ≥40.0 IU/L, **or**<br>• Surgically sterile including hysterectomy, bilateral oophorectomy, and/or tubal ligation, **or**<br>• Women of childbearing potential willing to use an acceptable method(s) of birth control during the study and for 30 days after the end of treatment, including:<br>o oral, topical, injectable, or implantable birth control medications,<br>o placement of an intrauterine device with or without hormones,<br>o barrier methods including condom or occlusive cap with spermicidal foam or spermicidal jelly,<br>o vasectomized male partner who is the sole partner for this patient,<br>o true abstinence that is in line with the preferred and usual lifestyle of the patient	Pregnancy is exclusion criterion; *other information not available*
Fasting LDL-C ≥100 mg/dL (2.6 mmol/L) at Week -5 (Visit S1) while taking stable (4 weeks prior to Visit S1) and optimized background LDL-C-lowering therapies that may include very low dose statin (see definition above), ezetimibe, niacin, bile acid resins, fibrates, and/or PCSK9 inhibitors	Baseline LDL-C ≥2.6 mmol/L

History of, or at high risk for, CVD including documented evidence of one or more of the following:	
a. Documented history of CVD (ie, secondary prevention)	
• CAD, defined by:	
- MI (either ST-elevation MI or non-ST-elevation MI) occurring >90 days prior to screening, **or**	- MI ever, inclusion because of MI
- Percutaneous coronary or surgical coronary revascularization, occurring >90 days prior to screening, **or**	- CABG or PCI ever, inclusion because of CABG or PCI
- Angiographic stenosis of ≥50% in a least 1 major coronary artery (native or graft vessel), as documented by selective coronary angiography or computed tomography angiography, **or**	- Inclusion because of ≥1 vessel disease on coronary angiography
• Symptomatic PAD, defined by:	
- Peripheral vascular disease with symptoms of claudication or resting limb ischemia with either ABI <0.9 or angiogram (including CTA) showing ≥50% stenosis, **or**	- Inclusion because of Fontaine classification ≥II (confirmed by ABI ≤0.90 at rest and/or ≥20% post-exercise decrease)
- Peripheral arterial revascularization (surgical or percutaneous), occurring >90 days prior to screening, **or**	- History of lower extremity bypass surgery or percutaneous transluminal angioplasty revascularization
- AAA confirmed by imaging or aortic aneurysm repair, occurring >90 days prior to screening, **or**	- Inclusion because of AAA, AAA ever, abdominal aorta diameter >3 cm on ultrasound at baseline, history of surgical or endovascular AAA repair
- Lower extremity amputation due to peripheral vascular disease, occurring >90 days prior to screening, **or**	- History of lower extremity amputation for arterial vascular disease
• CeVD, defined by:	
- Ischemic stroke occurring >90 days prior to screening, **or**	- Ischemic stroke ever, inclusion because of cerebral infarction
- Carotid endarterectomy, carotid stenting, or >70% stenosis in a carotid artery determined by carotid ultrasound or angiogram, occurring >90 days prior to screening, **or**	- History of carotid surgery or angioplasty, carotid artery stenosis >70% on carotid duplex ultrasound at baseline
b. High risk for a CVD event (ie, high-risk primary prevention)	
• Reynolds Risk score >30% or a Systematic Coronary Risk Evaluation (SCORE) Risk score >7.5% over 10 years, **or**	- *Criterion omitted because both scores not intended for secondary prevention and the study population includes only patients with established CAD, CeVD, and PAD per UCC-SMART definitions*
• Coronary artery calcium score >400 Agatston units at any time in the past, **or**	- *Information not available*
• Patients with type 1 or type 2 DM, aged >65 years (women) or >60 years (men)	- Women aged >65 years at inclusion or men aged >60 years at inclusion and inclusion because of DM or DM in medical history (type 1 or type 2)

Exclusion criteria

Total fasting TG >500 mg/dL (5.6 mmol/L)	Baseline TG >5.6 mmol/L
Renal dysfunction or a glomerulopathy defined as either nephritic or nephrotic syndrome, including eGFR (using central laboratory determined MDRD formula) <30 mL/min/1.73 m^2	eGFR (calculated with MDRD formula) <30 mL/min/1.73 m^2
Forms of CVD that include any of the following:	

a) Recent (<90 days prior to or during screening) acute CVD events including, but not only TIA, MI, coronary revascularization, peripheral arterial revascularization, ischemic stroke, carotid endarterectomy, carotid stenting	- *Assumption nobody since inclusion is generally > 1 month after acute event*
b) Recent (<90 days of screening) unstable or symptomatic cardiac arrhythmia including any associated medication changes). Patients with stable well-controlled atrial arrhythmias will be allowed to participate in the study.	- *Information not available*
c) Patients with implantable pacemakers or automatic implantable cardioverter defibrillators may be considered if deemed by the investigator to be stable for >90 days prior to screening	- *Information not available*
d) NYHA Functional Classification Class IV heart failure	- *Information not available*
e) Uncontrolled hypertension, defined as mean sitting SBP ≥180 mmHg and/or DBP ≥110 mmHg	- Baseline SBP ≥180 mmHg and/or DBP ≥110 mmHg
f) Planned coronary revascularization (patient may rescreen 3 months post-procedure).	- *Information not available*
HbA1c ≥10%	Baseline HbA1c ≥10%
Uncontrolled hypothyroidism, including TSH >1.5x ULN	Baseline TSH >7.5 mIU/L; *ULN according to local laboratory cutoff value*
Liver disease or dysfunction, including:	
- Positive serology for hepatitis B surface antigen and/or hepatitis C antibodies at Week -4 (Visit S2), or	- *Information not available*
- ALT and/or AST ≥2.0x ULN at Week -5 (Visit S1)	- *Information not available*
Gastrointestinal conditions or procedures (including weight loss surgery; eg, Lap-Band® or gastric bypass) that may affect drug absorption	*Information not available*
Hematologic or coagulation disorders or a hemoglobin level <10 g/dL	Baseline hemoglobin level <6.2058 mmol/L; *other information not available*
Active malignancy, including those requiring surgery, chemotherapy, and/or radiation in the past 5 years. Nonmetastatic basal or squamous cell carcinoma of the skin and cervical carcinoma in situ are allowed.	*Information not available*
Unexplained CK >3x ULN (ie, not associated with recent trauma or physically strenuous activity)	*Information not available*
History within the last 2 years of drug, alcohol, amphetamine and derivatives, or cocaine abuse. Patients with amphetamine derivatives prescribed by and under the care of a health care practitioner can be enrolled after evaluation by the investigator.	Self-reported weekly alcohol intake >21 standard drinks; *other information not available*
Blood transfusion for any reason <30 days prior to randomization	*Information not available*
Use of any experimental or investigational drugs <30 days prior to screening or 5 half-lives, whichever is longer	*Information not available*

Randomization into another Phase 3 bempedoic acid clinical study	*Information not available*
Use of, or a plan to initiate, these prohibited therapies/supplements during the study:<br>- Mipomersen (must be stopped ≥6 months prior to Week -5 [Visit S1]), lomitapide or apheresis therapy (must be stopped ≥3 months prior to Week -5 [Visit S1])<br>- Red yeast rice (must be stopped ≥2 weeks prior to Week -5 [Visit S1])<br>- Statins are prohibited at average daily doses of rosuvastatin ≥5 mg, atorvastatin ≥10 mg, simvastatin ≥10 mg, lovastatin ≥20 mg, pravastatin ≥40 mg, fluvastatin ≥40 mg, or pitavastatin ≥2 mg	 - Use of mipomersen or lomitapide at baseline; *information on apheresis therapy not available*<br>- *Information not available*<br>- *Assumption everybody using statin therapy at baseline was not statin intolerant*
Planned initiation or dose adjustments of these allowed drugs prior to screening and during the clinical trial (stable use of these drugs is permitted):<br>- Statins are allowed only at average daily doses of rosuvastatin <5 mg, atorvastatin <10 mg, simvastatin <10 mg, lovastatin <20 mg, pravastatin <40 mg, fluvastatin <40 mg, or pitavastatin <2 mg (must be stable ≥4 weeks prior to Week -5 [Visit S1])<br>- Other lipid-regulating drugs or supplements must be stable ≥4 weeks prior to Week -5 [Visit S1])<br>- PCSK9 inhibitors (must be stable ≥12 weeks prior to Week -5 [Visit S1])	 - *Information not available*<br>- *Information not available*<br>- *Information not available*
Lack of adherence (ie, less than 80% of planned doses) with IMP (single-blind placebo) during the Run-in Period	*Information not available*
Lack of tolerance with IMP (single-blind placebo) during the Run-in Period	*Information not available*
A medical or situational (ie, geographical) finding that in the investigator's opinion may compromise the patient's safety or ability to complete the study	*Information not available*
An employee or contractor of the facility conducting the study, or a family member of the Principal Investigator, Co-Investigator, or Sponsor	*Information not available*
Pregnant, breastfeeding, or intending to become pregnant <30 days after study completion or last dose of IMP	Pregnancy is exclusion criterion; *other information not available*

SPARCL[15]

Inclusion criteria

Men and women (either postmenopausal, surgically sterile or using reliable birth control) aged ≥18 years	Age ≥18 years at inclusion; *other information not available*
Previously documented stroke (ischemic or hemorrhagic) or TIA 1–6 months prior to randomization	Stroke ever, inclusion because of TIA, cerebral infarction, hemorrhagic stroke, or ischemic retinal syndrome

LDL-C level ≥100 mg/dl (2.6 mmol/L) and ≤190 mg/dl (4.9 mmol/L)	Baseline LDL-C ≥2.6 mmol/L and ≤4.9 mmol/L; for participants using LLT at baseline, untreated values estimated by adjusting measured LDL-C levels to account for ~30% reduction due to LLT
A Modified Rankin Score of ≤3 (i.e., functionally independent)	Independent in most daily activities is inclusion criterion (Rankin scale ≤3)
**Exclusion criteria**	
Women who were pregnant or breastfeeding	Pregnancy is exclusion criterion; *information on breastfeeding not available*
History of CHD	MI, CABG, or PCI ever<br>Inclusion because of MI, angina pectoris with proven stenosis on coronary angiogram, ≥1 vessel disease on coronary angiography, stable coronary artery disease, or coronary syndrome requiring PCI or CABG
History of significant peripheral vascular disease	Fontaine classification ≥II, history of lower extremity bypass surgery, percutaneous transluminal angioplasty revascularization, or amputation for arterial vascular disease
History of atrial fibrillation	*Information not available*
History of prosthetic heart valves	*Information not available*
History of clinically significant mitral stenosis	*Information not available*
History of sick node dysfunction	*Information not available*
History of uncontrolled hypertension	Baseline SBP ≥180 mmHg and/or DBP ≥110 mmHg; *aligned with cutoff values from other trials*
History of stroke caused by a revascularization procedure or trauma (as qualifying entry event)	*Information not available*
History of subarachnoid hemorrhage (as qualifying entry event)	Inclusion because of subarachnoid hemorrhage; *other information not available*
History of known hypersensitivity to statins or bile-sequestering resins	*Information not available*
History of active liver disease or hepatic dysfunction, defined as AST or ALT ≥2x ULN	*Information not available*
History of hematologic conditions that may cause thrombus formation	*Information not available*
History of endarterectomy <1 month before randomization	*Information not available*
History of severe renal dysfunction or nephrotic syndrome	Baseline eGFR <20 mL/min/1.73m^2; *aligned with cutoff values from other trials, information on nephrotic syndrome not available*
CK ≥5x ULN	*Information not available*

Participation in another clinical study <30 days prior to screening for the present study	*Information not available*
Diseases or abnormalities that the investigator believed might compromise the patient's safety during the study	*Information not available*
Unreliability as a study participant, based on the investigator's prior knowledge of the patient, such as alcoholism, drug abuse, or psychiatric illness	*Information not available*
Use of any drugs known to affect lipid levels or immunosuppressive agents, azole antifungals or drugs associated with rhabdomyolysis in combination with statins	Use of ciclosporin, systemic corticosteroids, or azole antifungals at baseline; *other information not available*
Patients taking a lipid-lowering drug could be considered for screening following a 30-day wash-out period (except in the case of probucol, in which medication must have been discontinued for ≥6 months)	*Criterion omitted because LLT is now standard care for patients with established ASCVD*

TST[17]

Inclusion criteria

Patient with:	
a) recent (<3 months) documented cerebral infarction once the neurologic deficit is stabilized (investigator judgement)	- Ischemic stroke ever, inclusion because of cerebral infarction
b) **or** recent (<15 days) TIA with at least arm of leg motor deficit or speech disturbance lasting >10 min	- Inclusion because of TIA
c) **or** a TIA with documented ischemic lesion (MRI or CT) in the appropriate area corresponding to the symptoms occurred <3 months before	- Inclusion because of TIA
**And** documented atherosclerotic disease:	
a) presence of carotid atherosclerotic disease (on the basis of carotid duplex, CTA, MRA, XRA – only the report will be required to document atherosclerotic stenosis)	- Inclusion because of duplex ultrasound confirmed asymptomatic carotid artery stenosis, carotid artery stenosis on duplex ultrasound at baseline
b) **or** presence of atherosclerotic disease of the aortic arch ≥4 mm (detected by transesophageal echocardiography or CT angiography)	- *Information not available*
c) **or** presence of atherosclerotic disease of another cerebral artery (documented vertebral artery stenosis, basilar artery stenosis, other intracranial artery stenosis)	- *Information not available*
d) **or** past-history of symptomatic CAD (based on known history of MI, revascularization procedure or known documented CAD by coronary angiography)	- MI, CABG, or PCI ever,<br>Inclusion because of MI, angina pectoris with proven stenosis on coronary angiogram, ≥1 vessel disease on coronary angiography, or coronary syndrome requiring PCI or CABG
Rankin score <5	Independent in most daily activities is inclusion criterion (Rankin scale ≤3)
A clear indication of statin treatment following AHA/ASA, ANSM or South Korean recommendations	*Assumption everybody since statins are now standard care for patients with established ASCVD*
Age >18 years (France) ≥20 years (South Korea)	Age ≥18 years at inclusion

Under contraception in case of childbearing potential	*Information not available*
**Exclusion criteria**	
Cerebral infarction/TIA due to arterial dissection (as documented following the judgment of the investigator) or due to cardiac source of embolism without documented atherosclerotic disease (e.g. mitral stenosis or endomyocardial fibrosis, endocarditis) [a patient with atrial fibrillation, or with a history of MI, or with calcified aortic stenosis will be eligible if the above inclusion criteria are also met]	*Information not available*
Symptomatic hemorrhagic stroke (the mere presence of asymptomatic cerebral microbleeding on gradient echo imaging is not an exclusion criteria)	Inclusion because of hemorrhagic stroke or subarachnoid hemorrhage
Uncontrolled hypertension (investigator judgment)	Baseline SBP ≥180 mmHg and/or DBP ≥110 mmHg; *aligned with cutoff values from other trials*
Baseline LDL-C <100 mg/dL while not taking statin [a patient with baseline LDL-C equal to 65 mg/dL, while on statin treatment can be randomized, and, if he is randomized in the 100 ± 10 mg/dL arm, statin treatment will be down-titrated to reach this target level], or patient for whom intensification of treatment is not feasible [e.g. patients with a baseline LDL-C above 100 mg/dL while on maximum dosage of the more potent statin, such as atorvastatin 80 mg/day or rosuvastatin 20 mg/day]	Baseline LDL-C <2.6 mmol/L and no use of statins at baseline **or** baseline LDL-C <1.7 mmol/L and use of statins at baseline; *other information not available*
Follow-up visit impossible or anticipated bad compliance	*Information not available*
Concurrent disease that may interfere with evaluation of the primary end-point or that may prevent follow-up study visits	*Information not available*
Participation to another clinical trial	*Information not available*

If information on a criterion was unavailable in UCC-SMART, it was not applied in the analysis. UCC-SMART = Utrecht Cardiovascular Cohort–Second Manifestations of ARTerial disease; CHD = coronary heart disease; MI = myocardial infarction; PTCA = percutaneous transluminal coronary angioplasty; CABG = coronary artery bypass grafting; PCI = percutaneous coronary intervention; TIA = transient ischemic attack; ABI = ankle-brachial index; DM = diabetes mellitus; HMG-CoA = Hydroxymethylglutaryl-Coenzyme A; ASCVD = atherosclerotic cardiovascular disease; ALT = alanine aminotransferase; AST = aspartate aminotransferase; ULN = upper limit of normal; HbA1c = glycated hemoglobin; TSH = thyroid stimulating hormone; SBP = systolic blood pressure; CK = creatine kinase; LDL-C = low-density lipoprotein cholesterol; LLT = lipid-lowering therapy; ACS = acute coronary syndrome; ECG = electrocardiogram; PAD = peripheral artery disease; CeVD = cerebrovascular disease; CAD = coronary artery disease; TG = triglycerides; HIV = human immunodeficiency virus; CVD = cardiovascular disease; HDL-C = high-density lipoprotein cholesterol; hsCRP = high-sensitivity C-reactive protein; DBP = diastolic blood pressure; NYHA = New York Heart Association; PCSK9 = proprotein convertase subtilisin/kexin type 9; LLN = lower limit of normal; eGFR = estimated glomerular filtration rate; MDRD = Modification of Diet in Renal Disease; IMP = Investigational Medicinal Product; CT = computed tomography.

† In the trial protocol defined as simvastatin >40 mg, atorvastatin ≥40 mg, all doses of rosuvastatin and ezetimibe co-administered with any dose of any statin. All other chronic prescription lipid-lowering therapies were considered less potent than simvastatin 40 mg daily, and subjects on these therapies could be enrolled.

§ The initial trial protocol allowed TG levels ≥135 mg/dL (1.52 mmol/L) by permitting a 10% deviation below the target lower limit. In May 2013, a study amendment raised the minimum fasting TG level from ≥150 mg/dL (1.69 mmol/L) to ≥200 mg/dL (2.26 mmol/L) to increase enrolment of patients with TG ≥200 mg/dL. For the present study, the original lower TG limit of ≥135 mg/dL (1.52 mmol/L) was used.

Supplementary Figure 1. Application of trial eligibility criteria to UCC-SMART patients with cerebrovascular disease

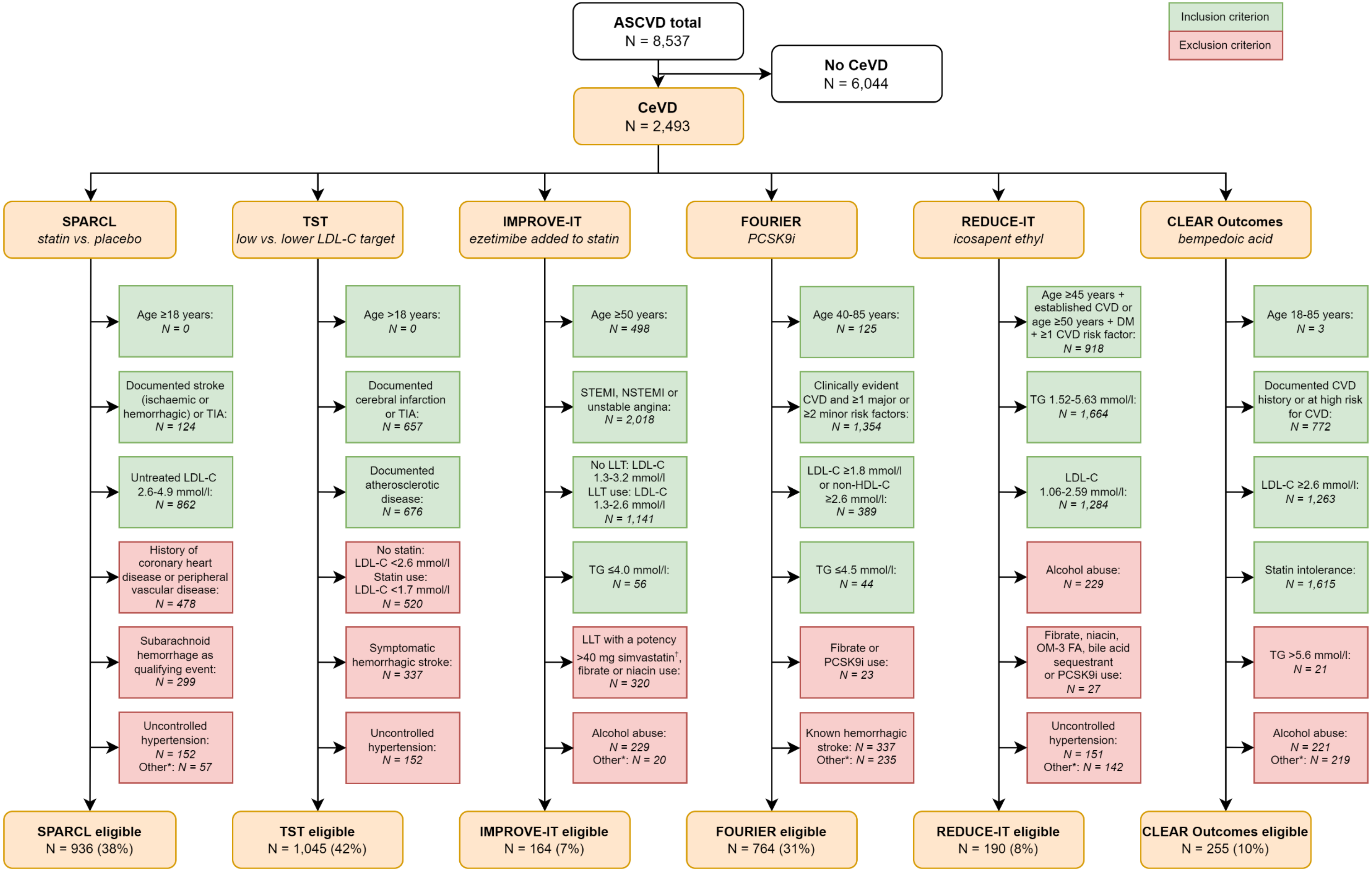

Numbers in rectangles represent the number of patients not fulfilling that eligibility criterion. UCC-SMART = Utrecht Cardiovascular Cohort–Second Manifestations of ARTerial disease; ASCVD = atherosclerotic cardiovascular disease; N = number; CeVD = cerebrovascular disease; SPARCL = Stroke Prevention by Aggressive Reduction in Cholesterol Levels trial; TST = Treat Stroke to Target trial; IMPROVE-IT = IMProved Reduction of Outcomes: Vytorin Efficacy Intervention Trial; FOURIER = Further Cardiovascular Outcomes Research with PCSK9 Inhibition in Subjects with Elevated Risk; PCSK9i = proprotein convertase subtilisin/kexin type 9 inhibitor; REDUCE-IT = Reduction of Cardiovascular Events with Icosapent Ethyl–Intervention Trial; CLEAR Outcomes = Cholesterol Lowering via Bempedoic Acid, an ACL-Inhibiting Regimen Outcomes trial; TIA = transient ischemic attack; LDL-C = low-density lipoprotein cholesterol; STEMI = ST-elevation myocardial infarction; NSTEMI = non-ST-elevation myocardial infarction; LLT = lipid-lowering therapy; TG = triglycerides; non-HDL-C = non-high-density lipoprotein cholesterol; DM = diabetes mellitus; OM-3 FA = omega-3 fatty acid.

* Eligibility criteria under 'Other' varied between trials. SPARCL: severe renal dysfunction and concomitant medications. IMPROVE-IT: creatinine clearance <30 ml/min and concomitant medications. FOURIER: uncontrolled hypertension, TSH <LLN or >1.5x ULN and estimated glomerular filtrate rate <20 ml/min/1.73m2. REDUCE-IT: HbA1c >10%, creatinine clearance <30 ml/min and concomitant medications. CLEAR Outcomes: estimated glomerular filtrate rate <30 ml/min/1.73m2, SBP ≥180 mmHg or DBP ≥110 mmHg, HbA1c ≥10%, TSH >1.5x ULN and hemoglobin <10 g/dl.

† In the trial protocol defined as simvastatin >40 mg, atorvastatin ≥40 mg, all doses of rosuvastatin and ezetimibe co-administered with any dose of any statin.

Supplementary Figure 2. Application of trial eligibility criteria to UCC-SMART patients with peripheral artery disease.

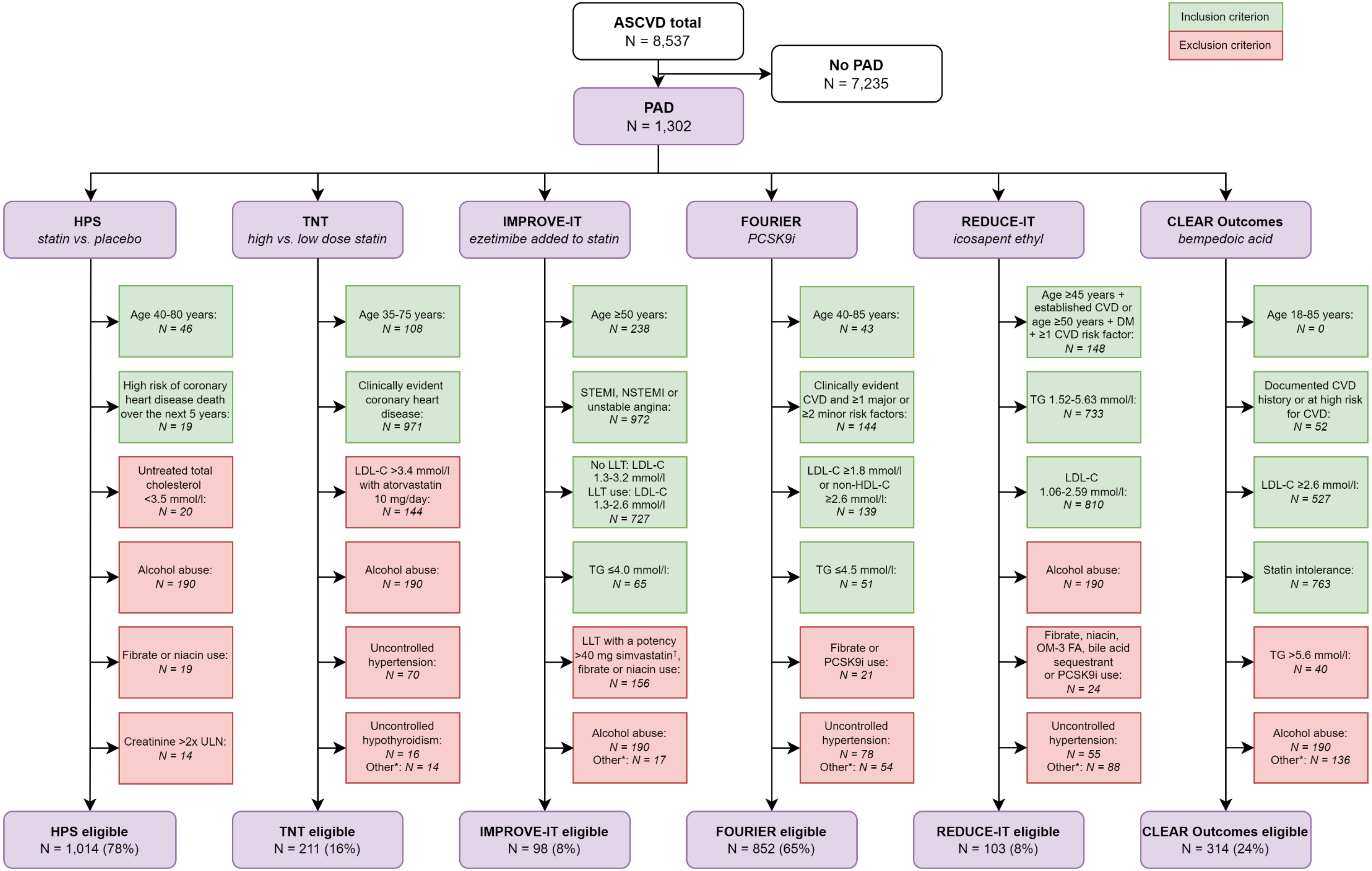

Numbers in rectangles represent the number of patients not fulfilling that eligibility criterion. UCC-SMART = Utrecht Cardiovascular Cohort–Second Manifestations of ARTerial disease; ASCVD = atherosclerotic cardiovascular disease; N = number; PAD = peripheral artery disease; HPS = Heart Protection Study; TNT = Treating to New Targets trial; IMPROVE-IT = IMProved Reduction of Outcomes: Vytorin Efficacy Intervention Trial; FOURIER = Further Cardiovascular Outcomes Research with PCSK9 Inhibition in Subjects with Elevated Risk; PCSK9i = proprotein convertase subtilisin/kexin type 9 inhibitor; REDUCE-IT = Reduction of Cardiovascular Events with Icosapent Ethyl–Intervention Trial; CLEAR Outcomes = Cholesterol Lowering via Bempedoic Acid, an ACL-Inhibiting Regimen Outcomes trial; ULN = upper limit of normal; LDL-C = low-density lipoprotein cholesterol; STEMI = ST-elevation myocardial infarction; NSTEMI = non-ST-elevation myocardial infarction; LLT = lipid-lowering therapy; TG = triglycerides; non-HDL-C = non-high-density lipoprotein cholesterol; DM = diabetes mellitus; OM-3 FA = omega-3 fatty acid.

* Eligibility criteria under 'Other' varied between trials. TNT: HbA1c >10%. IMPROVE-IT: creatinine clearance <30 ml/min and concomitant medications. FOURIER: known hemorrhagic stroke at any time, TSH <LLN or >1.5x ULN and estimated glomerular filtrate rate <20 ml/min/1.73m^2. REDUCE-IT: HbA1c >10%, creatinine clearance <30 ml/min and concomitant medications. CLEAR Outcomes: estimated glomerular filtrate rate <30 ml/min/1.73m^2, SBP ≥180 mmHg or DBP ≥110 mmHg, HbA1c ≥10%, TSH >1.5x ULN and hemoglobin <10 g/dl.

† In the trial protocol defined as simvastatin >40 mg, atorvastatin ≥40 mg, all doses of rosuvastatin and ezetimibe co-administered with any dose of any statin.

Supplementary Table 2. Clinical characteristics of trial-eligible and trial-ineligible UCC-SMART patients with cerebrovascular disease.

	**SPARCL** *statin vs. placebo*		**TST** *low vs. lower LDL-C target*		**IMPROVE-IT** *ezetimibe added to statin*		**FOURIER** *PCSK9i*		**REDUCE-IT** *icosapent ethyl*		**CLEAR Outcomes** *bempedoic acid*	
	Ineligible *N = 1557*	Eligible *N = 936*	Ineligible *N = 1448*	Eligible *N = 1045*	Ineligible *N = 2329*	Eligible *N = 164*	Ineligible *N = 1729*	Eligible *N = 764*	Ineligible *N = 2303*	Eligible *N = 190*	Ineligible *N = 2238*	Eligible *N = 255*
**Patient characteristics**												
Age (years)	60 ± 11	59 ± 11*	58 ± 12	63 ± 10*	59 ± 11	66 ± 7*	59 ± 12	62 ± 10*	59 ± 11	62 ± 9*	60 ± 11	59 ± 13
Male sex	940 (60)	571 (61)	785 (54)	726 (69)*	1383 (59)	128 (78)*	1004 (58)	507 (66)*	1374 (60)	137 (72)*	1353 (60)	158 (62)
Current smoker	439 (28)	289 (31)	390 (27)	338 (32)*	694 (30)	34 (21)*	448 (26)	280 (37)*	673 (29)	55 (29)	655 (29)	72 (28)
**Medical history**												
Coronary artery disease	488 (31)	6 (1)*	195 (13)	299 (29)*	330 (14)	164 (100)*	214 (12)	280 (37)*	419 (18)	75 (40)*	451 (20)	43 (17)
Peripheral artery disease	147 (9)	0 (0)*	72 (5)	75 (7)*	128 (6)	19 (11)*	54 (3)	93 (12)*	130 (6)	17 (9)	127 (6)	20 (8)
Abdominal aortic aneurysm	71 (5)	24 (3)*	44 (3)	51 (5)*	85 (4)	10 (6)	53 (3)	42 (6)*	82 (4)	13 (7)	76 (3)	19 (7)*
Diabetes mellitus	273 (18)	104 (11)*	200 (14)	177 (17)*	336 (14)	41 (25)*	218 (13)	159 (21)*	318 (14)	59 (31)*	337 (15)	40 (16)
10-year risk of recurrent CVD[a] (%)	20 [14-32]	16 [12-23]*	16 [11-24]	22 [15-33]*	18 [12-27]	30 [21-41]*	17 [11-24]	25 [17-35]*	18 [13-28]	23 [16-34]*	18 [12-28]	22 [15-34]*
**Medication**												
Blood pressure-lowering therapy	1035 (66)	547 (58)*	828 (57)	754 (72)*	1444 (62)	139 (85)*	1012 (59)	571 (75)*	1426 (62)	157 (83)*	1442 (64)	141 (55)*
Glucose-lowering therapy	188 (12)	63 (7)*	133 (9)	119 (11)	225 (10)	27 (16)*	146 (8)	105 (14)*	214 (9)	37 (20)*	230 (10)	22 (9)
Antithrombotic therapy	1092 (70)	735 (79)*	948 (66)	878 (84)*	1684 (72)	143 (87)*	1195 (69)	631 (83)*	1666 (72)	161 (85)*	1657 (74)	170 (67)*
Lipid-lowering therapy	1003 (64)	636 (68)	796 (55)	842 (81)*	1502 (65)	136 (83)*	1057 (61)	582 (76)*	1464 (64)	175 (92)*	1621 (72)	17 (7)*
Statins	983 (63)	632 (68)*	785 (54)	829 (79)*	1479 (63)	136 (83)*	1043 (60)	572 (75)*	1441 (63)	174 (91)*	1615 (72)	0 (0)*
Ezetimibe	83 (5)	16 (2)*	53 (4)	45 (4)	92 (4)	6 (4)	59 (3)	39 (5)	87 (4)	11 (6)	84 (4)	14 (5)
**Physical examination**												
Systolic blood pressure (mmHg)	142 ± 23	136 ± 17*	141 ± 24	138 ± 17*	140 ± 22	139 ± 18	141 ± 23	137 ± 18*	140 ± 22	137 ± 18	140 ± 22	139 ± 17
Diastolic blood pressure (mmHg)	82 ± 13	81 ± 10*	83 ± 13	80 ± 10*	82 ± 12	79 ± 10*	83 ± 13	80 ± 10*	82 ± 12	79 ± 10*	82 ± 12	81 ± 10
Body mass index (kg/m²)	27 ± 4	27 ± 4	27 ± 4	27 ± 4	27 ± 4	27 ± 4*	27 ± 4	27 ± 4	27 ± 4	28 ± 4*	27 ± 4	27 ± 5
**Laboratory results**												
Total cholesterol (mmol/l)	4.7 ± 1.3	4.8 ± 0.8	4.6 ± 1.3	4.9 ± 1.0*	4.8 ± 1.2	3.9 ± 0.6*	4.7 ± 1.2	4.8 ± 1.0*	4.8 ± 1.2	4.1 ± 0.5*	4.6 ± 1.2	5.6 ± 0.9*
HDL-C (mmol/l)	1.3 ± 0.4	1.3 ± 0.4*	1.3 ± 0.4	1.3 ± 0.4*	1.3 ± 0.4	1.2 ± 0.3*	1.4 ± 0.4	1.3 ± 0.4*	1.3 ± 0.4	1.1 ± 0.3*	1.3 ± 0.4	1.3 ± 0.4
LDL-C (mmol/l)	3.4 ± 1.4[b]	3.5 ± 0.6*[b]	2.6 ± 1.1	2.9 ± 0.9*	2.8 ± 1.0	2.1 ± 0.4*	2.7 ± 1.1	2.9 ± 0.9*	2.8 ± 1.0	2.0 ± 0.4*	2.6 ± 1.0	3.6 ± 0.8*
Triglycerides (mmol/l)	1.3 [0.9-1.8]	1.2 [0.9-1.7]*	1.2 [0.8-1.7]	1.3 [1.0-1.8]*	1.2 [0.9-1.8]	1.3 [1.0-1.8]	1.2 [0.9-1.7]	1.4 [1.0-1.9]*	1.2 [0.9-1.6]	2.0 [1.7-2.6]*	1.2 [0.9-1.7]	1.4 [1.1-1.8]*
HbA1c (%)	5.6 [5.4-6.0]	5.6 [5.4-5.9]*	5.6 [5.3-5.9]	5.7 [5.4-6.0]*	5.6 [5.4-5.9]	5.7 [5.4-6.1]*	5.6 [5.3-5.9]	5.7 [5.4-6.0]*	5.6 [5.4-5.9]	5.9 [5.5-6.4]*	5.6 [5.4-5.9]	5.6 [5.4-5.9]
eGFR (ml/min/1.73 m²)	78 ± 19	79 ± 17*	81 ± 19	75 ± 18*	79 ± 19	71 ± 16*	80 ± 18	75 ± 18*	78 ± 19	75 ± 17*	78 ± 18	78 ± 19

Data are presented as count (%) for categorical variables, median [interquartile range] for 10-year risk of recurrent CVD, triglycerides and HbA1c and mean ± standard deviation for other continuous variables.
UCC-SMART = Utrecht Cardiovascular Cohort–Second Manifestations of ARTerial disease; SPARCL = Stroke Prevention by Aggressive Reduction in Cholesterol Levels trial; TST = Treat Stroke to Target trial; IMPROVE-IT = IMProved Reduction of Outcomes: Vytorin Efficacy Intervention Trial; FOURIER = Further Cardiovascular Outcomes Research with PCSK9 Inhibition in Subjects with Elevated Risk; PCSK9i = proprotein convertase subtilisin/kexin type 9 inhibitor; REDUCE-IT = Reduction of Cardiovascular Events with Icosapent Ethyl–Intervention Trial; CLEAR Outcomes = Cholesterol Lowering via Bempedoic Acid, an ACL-Inhibiting Regimen Outcomes trial; N = number; CVD = cardiovascular disease; HDL-C = high-density lipoprotein cholesterol; LDL-C = low-density lipoprotein cholesterol; HbA1c = glycated hemoglobin; eGFR = estimated glomerular filtration rate (calculated with Chronic Kidney Disease Epidemiology Collaboration [CKD-EPI] formula).

* Statistically significant difference between trial-eligible and ineligible patients (*p*-value <0.05).

[a] Calculated using the SMART2 risk score.[26]

[b] Recalculated to untreated levels.

Supplementary Table 3. Clinical characteristics of trial-eligible and trial-ineligible UCC-SMART patients with peripheral artery disease.

	**HPS** *statin vs. placebo*		**TNT** *high vs. low dose statin*		**IMPROVE-IT** *ezetimibe added to statin*		**FOURIER** *PCSK9i*		**REDUCE-IT** *icosapent ethyl*		**CLEAR Outcomes** *bempedoic acid*	
	Ineligible *N = 288*	Eligible *N = 1014*	Ineligible *N = 1091*	Eligible *N = 211*	Ineligible *N = 1204*	Eligible *N = 98*	Ineligible *N = 450*	Eligible *N = 852*	Ineligible *N = 1199*	Eligible *N = 103*	Ineligible *N = 988*	Eligible *N = 314*
**Patient characteristics**												
Age (years)	57 ± 12	61 ± 10*	59 ± 11	62 ± 8*	59 ± 11	64 ± 7*	58 ± 12	61 ± 10*	60 ± 11	61 ± 8	60 ± 10	59 ± 11
Male sex	224 (78)	649 (64)*	709 (65)	164 (78)*	795 (66)	78 (80)*	273 (61)	600 (70)*	796 (66)	77 (74)	680 (69)	193 (61)*
Current smoker	147 (51)	495 (49)	556 (51)	86 (41)*	613 (51)	29 (29)*	173 (38)	470 (55)*	590 (49)	52 (50)	473 (48)	169 (54)
**Medical history**												
Coronary artery disease	72 (25)	269 (27)	130 (12)	211 (100)*	243 (20)	98 (100)*	104 (23)	237 (28)	294 (25)	47 (46)*	295 (30)	46 (15)*
Cerebrovascular disease	29 (10)	118 (12)	113 (10)	34 (16)*	128 (11)	19 (19)*	54 (12)	93 (11)	130 (11)	17 (16)	127 (13)	20 (6)*
Abdominal aortic aneurysm	30 (11)	88 (9)	88 (8)	30 (14)*	105 (9)	13 (13)	37 (8)	81 (10)	102 (9)	16 (15)	94 (10)	24 (8)
Diabetes mellitus	51 (18)	221 (22)	215 (20)	57 (27)*	239 (20)	33 (34)*	101 (22)	172 (20)	234 (20)	38 (37)*	230 (23)	42 (13)*
10-year risk of recurrent CVD[a] (%)	21 [15-32]	22 [16-32]	21 [15-30]	29 [20-44]*	22 [15-31]	29 [21-44]*	19 [13-29]	23 [17-33]*	22 [15-32]	25 [18-36]*	22 [15-32]	23 [16-32]
**Medication**												
Blood pressure-lowering therapy	162 (56)	604 (59)	583 (53)	182 (86)*	682 (57)	83 (85)*	271 (60)	494 (58)	685 (57)	80 (77)*	649 (66)	116 (37)*
Glucose-lowering therapy	28 (10)	140 (14)*	129 (12)	39 (19)*	145 (12)	23 (23)*	61 (14)	107 (13)	143 (12)	25 (24)*	140 (14)	28 (9)*
Antithrombotic therapy	177 (61)	675 (67)	680 (62)	172 (81)*	776 (64)	76 (78)*	300 (67)	552 (65)	776 (65)	76 (73)	707 (72)	145 (46)*
Lipid-lowering therapy	163 (57)	618 (61)	620 (57)	161 (76)*	704 (58)	77 (79)*	296 (66)	485 (57)*	692 (58)	89 (86)*	773 (78)	8 (3)*
Statins	157 (55)	606 (60)	607 (56)	156 (74)*	687 (57)	76 (78)*	285 (63)	478 (56)*	674 (56)	89 (86)*	763 (77)	0 (0 *
Ezetimibe	19 (6)	62 (6)	59 (5)	22 (10)*	73 (6)	8 (8)	35 (8)	46 (5)	73 (6)	8 (8)	76 (8)	5 (2)*
**Physical examination**												
Systolic blood pressure (mmHg)	142 ± 20	144 ± 22	144 ± 22	139 ± 17*	143 ± 22	140 ± 18	148 ± 27	141 ± 17*	143 ± 22	143 ± 18	144 ± 22	139 ± 18*
Diastolic blood pressure (mmHg)	81 ± 11	81 ± 11	82 ± 11	77 ± 10*	82 ± 11	78 ± 11*	84 ± 13	80 ± 10*	82 ± 12	80 ± 9	82 ± 12	80 ± 10
Body mass index (kg/m²)	26 ± 4	27 ± 4*	26 ± 4	28 ± 4*	26 ± 4	28 ± 4*	26 ± 4	27 ± 4	26 ± 4	29 ± 4*	27 ± 4	26 ± 4
**Laboratory results**												
Total cholesterol (mmol/l)	5.9 ± 1.5[b]	6.0 ± 1.3[b]	5.1 ± 1.2	4.5 ± 1.0*	5.1 ± 1.2	4.1 ± 0.6*	4.8 ± 1.4	5.2 ± 1.0*	5.1 ± 1.2	4.2 ± 0.6*	4.8 ± 1.2	5.8 ± 0.9*
HDL-C (mmol/l)	1.3 ± 0.4	1.2 ± 0.4	1.3 ± 0.4	1.2 ± 0.4*	1.3 ± 0.4	1.2 ± 0.3*	1.3 ± 0.4	1.2 ± 0.4*	1.3 ± 0.4	1.1 ± 0.3*	1.3 ± 0.4	1.2 ± 0.4
LDL-C (mmol/l)	2.9 ± 1.1	3.0 ± 1.0	2.5 ± 0.8[c]	2.2 ± 0.6*[c]	3.0 ± 1.1	2.1 ± 0.4*	2.5 ± 1.1	3.2 ± 0.9*	3.0 ± 1.1	2.0 ± 0.4*	2.7 ± 1.0	3.8 ± 0.8*
Triglycerides (mmol/l)	1.5 [1.0-2.3]	1.5 [1.1-2.1]	1.4 [1.0-2.1]	1.6 [1.2-2.4]*	1.5 [1.1-2.2]	1.5 [1.0-2.2]	1.4 [1.0-2.2]	1.5 [1.1-2.1]	1.4 [1.0-2.0]	2.3 [1.9-2.8]*	1.4 [1.0-2.2]	1.6 [1.1-2.1]
HbA1c (%)	5.7 [5.4-6.0]	5.7 [5.5-6.1]*	5.7 [5.4-6.1]	5.8 [5.5-6.4]*	5.7 [5.4-6.1]	5.9 [5.6-6.4]*	5.7 [5.4-6.1]	5.7 [5.5-6.1]	5.7 [5.4-6.1]	5.9 [5.6-6.7]*	5.7 [5.5-6.2]	5.7 [5.4-6.0]*
eGFR (ml/min/1.73 m²)	80 ± 24	77 ± 18*	79 ± 20	74 ± 18*	78 ± 20	71 ± 18*	77 ± 22	78 ± 18	78 ± 20	76 ± 19	78 ± 20	79 ± 17

Data are presented as count (%) for categorical variables, median [interquartile range] for 10-year risk of recurrent CVD, triglycerides and HbA1c and mean ± standard deviation for other continuous variables.
UCC-SMART = Utrecht Cardiovascular Cohort–Second Manifestations of ARTerial disease; SPARCL = Stroke Prevention by Aggressive Reduction in Cholesterol Levels trial; TST = Treat Stroke to Target trial; IMPROVE-IT = IMProved Reduction of Outcomes: Vytorin Efficacy Intervention Trial; FOURIER = Further Cardiovascular Outcomes Research with PCSK9 Inhibition in Subjects with Elevated Risk; PCSK9i = proprotein convertase subtilisin/kexin type 9 inhibitor; REDUCE-IT = Reduction of Cardiovascular Events with Icosapent Ethyl–Intervention Trial; CLEAR Outcomes = Cholesterol Lowering via Bempedoic Acid, an ACL-Inhibiting Regimen Outcomes trial; N = number; CVD = cardiovascular disease; HDL-C = high-density lipoprotein cholesterol; LDL-C = low-density lipoprotein cholesterol; HbA1c = glycated hemoglobin; eGFR = estimated glomerular filtration rate (calculated with Chronic Kidney Disease Epidemiology Collaboration [CKD-EPI] formula).
* Statistically significant difference between trial-eligible and ineligible patients (p-value <0.05).
[a] Calculated using the SMART2 risk score.[26]
[b] Recalculated to untreated levels.
[c] Recalculated to levels under atorvastatin 10 mg/day.

Supplementary Table 4. Rate ratios for recurrent CV events and all-cause mortality for trial-eligible compared to trial-ineligible UCC-SMART patients.

Trial	Coronary artery disease patients N = 5,673		Cerebrovascular disease patients N = 2,493		Peripheral artery disease patients N = 1,302	
	**Recurrent CV events** N = 1,104	**All-cause mortality** N = 1,309	**Recurrent CV events** N = 479	**All-cause mortality** N = 655	**Recurrent CV events** N = 317	**All-cause mortality** N = 515
**HPS**	0.81 (0.69–0.95)	0.80 (0.70–0.93)	N/A	N/A	0.99 (0.75–1.30)	0.87 (0.71–1.07)
**TNT**	0.70 (0.62–0.79)	0.60 (0.54–0.67)	N/A	N/A	1.66 (1.27–2.16)	1.29 (1.04–1.61)
**IMPROVE-IT**	1.00 (0.88–1.13)	1.06 (0.95–1.18)	2.12 (1.60–2.81)	1.94 (1.52–2.48)	1.94 (1.40–2.70)	1.44 (1.08–1.93)
**FOURIER**	1.55 (1.37–1.75)	1.49 (1.33–1.66)	1.93 (1.60–2.32)	1.59 (1.36–1.86)	1.04 (0.81–1.32)	1.06 (0.88–1.29)
**REDUCE-IT**	0.93 (0.78–1.11)	0.92 (0.78–1.08)	1.45 (1.07–1.97)	1.29 (0.98–1.69)	1.19 (0.81–1.75)	0.92 (0.66–1.29)
**CLEAR Outcomes**	1.26 (1.05–1.51)	1.29 (1.09–1.53)	1.29 (0.98–1.69)	1.21 (0.96–1.53)	0.83 (0.64–1.08)	0.92 (0.75–1.12)
**SPARCL**	N/A	N/A	0.68 (0.56–0.83)	0.70 (0.59–0.82)	N/A	N/A
**TST**	N/A	N/A	1.68 (1.40–2.01)	1.42 (1.22–1.66)	N/A	N/A

Rate ratios are presented with 95% confidence intervals. UCC-SMART = Utrecht Cardiovascular Cohort – Second Manifestations of ARTerial disease; CV = cardiovascular; N = number; N/A = not applicable; HPS = Heart Protection Study; TNT = Treating to New Targets trial; IMPROVE-IT = IMProved Reduction of Outcomes: Vytorin Efficacy Internation Trial; FOURIER = Further Cardiovascular Outcomes Research with PCSK9 Inhibition in Subjects with Elevated Risk; PCSK9i = proprotein convertase subtilisin/kexin type 9 inhibitor; REDUCE-IT = Reduction of Cardiovascular Events with Icosapent Ethyl-Intervention Trial; CLEAR Outcomes = Cholesterol Lowering via Bempedoic Acid, an ACL-Inhibiting Regimen Outcomes trial; SPARCL = Stroke Prevention by Aggressive Reduction in Cholesterol Levels trial; TST = Treat Stroke to Target trial.

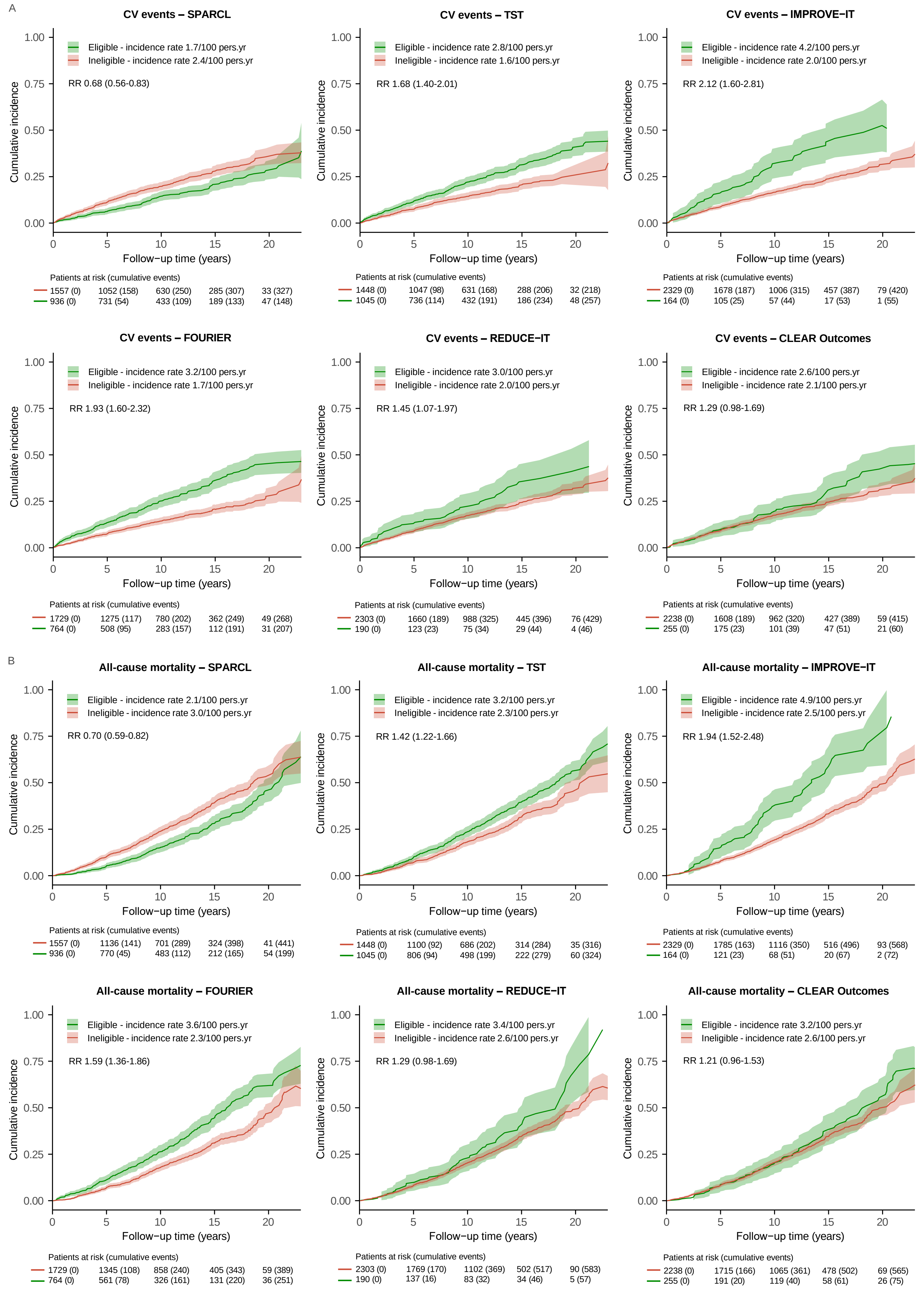
A
CV events – SPARCL
Eligible - incidence rate 1.7/100 pers.yr
Ineligible - incidence rate 2.4/100 pers.yr
RR 0.68 (0.56-0.83)
Cumulative incidence
Follow−up time (years)
Patients at risk (cumulative events)
1557 (0) 1052 (158) 630 (250) 285 (307) 33 (327)
936 (0) 731 (54) 433 (109) 189 (133) 47 (148)
CV events – TST
Eligible - incidence rate 2.8/100 pers.yr
Ineligible - incidence rate 1.6/100 pers.yr
RR 1.68 (1.40-2.01)
Patients at risk (cumulative events)
1448 (0) 1047 (98) 631 (168) 288 (206) 32 (218)
1045 (0) 736 (114) 432 (191) 186 (234) 48 (257)
CV events – IMPROVE−IT
Eligible - incidence rate 4.2/100 pers.yr
Ineligible - incidence rate 2.0/100 pers.yr
RR 2.12 (1.60-2.81)
Patients at risk (cumulative events)
2329 (0) 1678 (187) 1006 (315) 457 (387) 79 (420)
164 (0) 105 (25) 57 (44) 17 (53) 1 (55)
CV events – FOURIER
Eligible - incidence rate 3.2/100 pers.yr
Ineligible - incidence rate 1.7/100 pers.yr
RR 1.93 (1.60-2.32)
Patients at risk (cumulative events)
1729 (0) 1275 (117) 780 (202) 362 (249) 49 (268)
764 (0) 508 (95) 283 (157) 112 (191) 31 (207)
CV events – REDUCE−IT
Eligible - incidence rate 3.0/100 pers.yr
Ineligible - incidence rate 2.0/100 pers.yr
RR 1.45 (1.07-1.97)
Patients at risk (cumulative events)
2303 (0) 1660 (189) 988 (325) 445 (396) 76 (429)
190 (0) 123 (23) 75 (34) 29 (44) 4 (46)
CV events – CLEAR Outcomes
Eligible - incidence rate 2.6/100 pers.yr
Ineligible - incidence rate 2.1/100 pers.yr
RR 1.29 (0.98-1.69)
Patients at risk (cumulative events)
2238 (0) 1608 (189) 962 (320) 427 (389) 59 (415)
255 (0) 175 (23) 101 (39) 47 (51) 21 (60)
B
All-cause mortality – SPARCL
Eligible - incidence rate 2.1/100 pers.yr
Ineligible - incidence rate 3.0/100 pers.yr
RR 0.70 (0.59-0.82)
Patients at risk (cumulative events)
1557 (0) 1136 (141) 701 (289) 324 (398) 41 (441)
936 (0) 770 (45) 483 (112) 212 (165) 54 (199)
All-cause mortality – TST
Eligible - incidence rate 3.2/100 pers.yr
Ineligible - incidence rate 2.3/100 pers.yr
RR 1.42 (1.22-1.66)
Patients at risk (cumulative events)
1448 (0) 1100 (92) 686 (202) 314 (284) 35 (316)
1045 (0) 806 (94) 498 (199) 222 (279) 60 (324)
All-cause mortality – IMPROVE−IT
Eligible - incidence rate 4.9/100 pers.yr
Ineligible - incidence rate 2.5/100 pers.yr
RR 1.94 (1.52-2.48)
Patients at risk (cumulative events)
2329 (0) 1785 (163) 1116 (350) 516 (496) 93 (568)
164 (0) 121 (23) 68 (51) 20 (67) 2 (72)
All-cause mortality – FOURIER
Eligible - incidence rate 3.6/100 pers.yr
Ineligible - incidence rate 2.3/100 pers.yr
RR 1.59 (1.36-1.86)
Patients at risk (cumulative events)
1729 (0) 1345 (108) 858 (240) 405 (343) 59 (389)
764 (0) 561 (78) 326 (161) 131 (220) 36 (251)
All-cause mortality – REDUCE−IT
Eligible - incidence rate 3.4/100 pers.yr
Ineligible - incidence rate 2.6/100 pers.yr
RR 1.29 (0.98-1.69)
Patients at risk (cumulative events)
2303 (0) 1769 (170) 1102 (369) 502 (517) 90 (583)
190 (0) 137 (16) 83 (32) 34 (46) 5 (57)
All-cause mortality – CLEAR Outcomes
Eligible - incidence rate 3.2/100 pers.yr
Ineligible - incidence rate 2.6/100 pers.yr
RR 1.21 (0.96-1.53)
Patients at risk (cumulative events)
2238 (0) 1715 (166) 1065 (361) 478 (502) 69 (565)
255 (0) 191 (20) 119 (40) 58 (61) 26 (75)

Supplementary Figure 3. Cumulative incidence of recurrent CV events (A) and all-cause mortality (B) in trial-eligible and trial-ineligible UCC-SMART patients with cerebrovascular disease.

Rate ratios are presented with 95% confidence intervals for trial-eligible patients compared to trial-ineligible patients. UCC-SMART = Utrecht Cardiovascular Cohort–Second Manifestations of ARTerial disease; CV = cardiovascular; pers.yr = person-years; RR = rate ratio; SPARCL = Stroke Prevention by Aggressive Reduction in Cholesterol Levels trial; TST = Treat Stroke to Target trial; IMPROVE-IT = IMProved Reduction of Outcomes: Vytorin Efficacy Intervention Trial; FOURIER = Further Cardiovascular Outcomes Research with PCSK9 Inhibition in Subjects with Elevated Risk; PCSK9 = proprotein convertase subtilisin/kexin type 9; REDUCE-IT = Reduction of Cardiovascular Events with Icosapent Ethyl–Intervention Trial; CLEAR Outcomes = Cholesterol Lowering via Bempedoic Acid, an ACL-Inhibiting Regimen Outcomes trial.

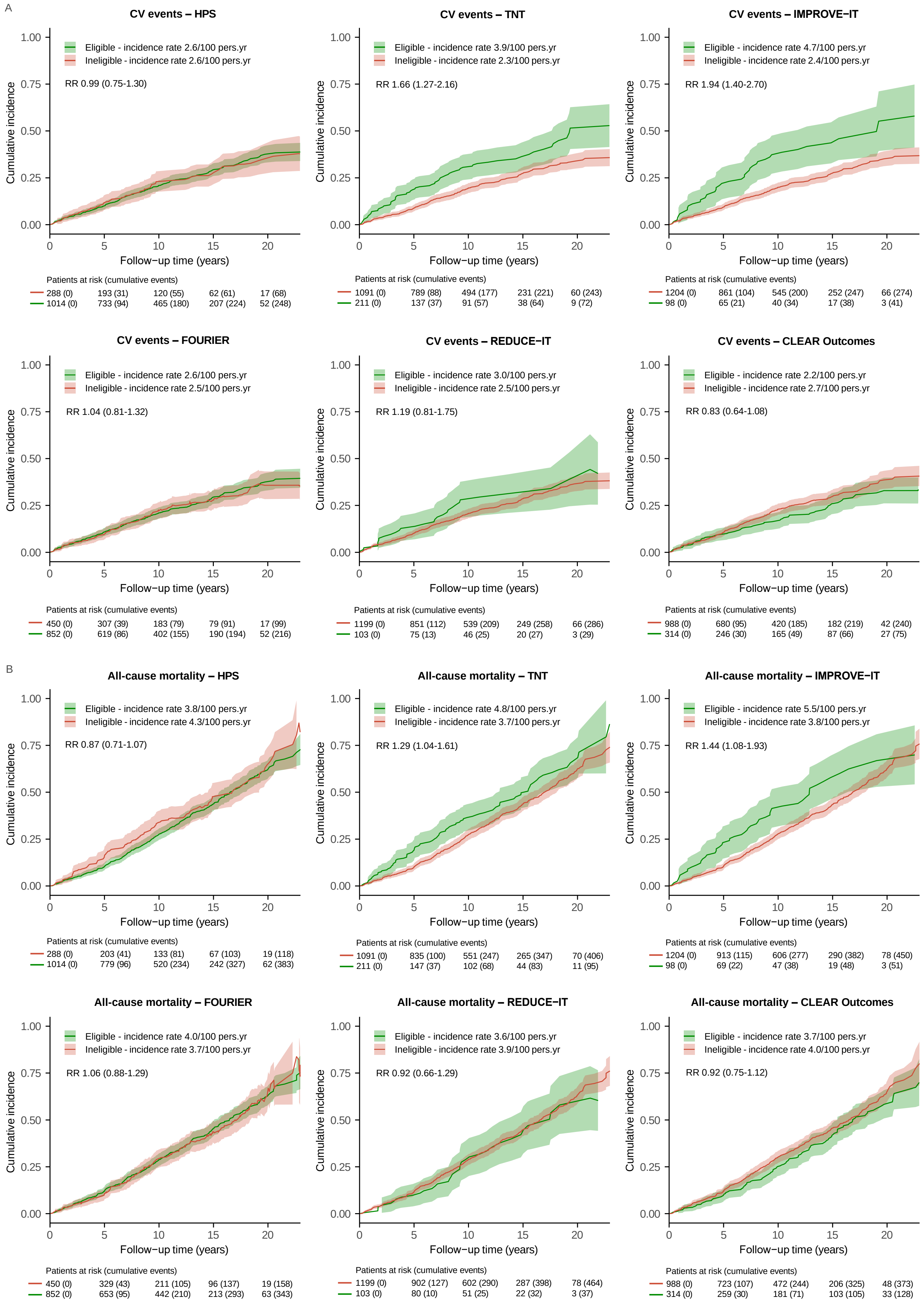

A
CV events – HPS
Eligible - incidence rate 2.6/100 pers.yr
Ineligible - incidence rate 2.6/100 pers.yr
RR 0.99 (0.75-1.30)
Patients at risk (cumulative events)
288 (0) 193 (31) 120 (55) 62 (61) 17 (68)
1014 (0) 733 (94) 465 (180) 207 (224) 52 (248)
CV events – TNT
Eligible - incidence rate 3.9/100 pers.yr
Ineligible - incidence rate 2.3/100 pers.yr
RR 1.66 (1.27-2.16)
Patients at risk (cumulative events)
1091 (0) 789 (88) 494 (177) 231 (221) 60 (243)
211 (0) 137 (37) 91 (57) 38 (64) 9 (72)
CV events – IMPROVE-IT
Eligible - incidence rate 4.7/100 pers.yr
Ineligible - incidence rate 2.4/100 pers.yr
RR 1.94 (1.40-2.70)
Patients at risk (cumulative events)
1204 (0) 861 (104) 545 (200) 252 (247) 66 (274)
98 (0) 65 (21) 40 (34) 17 (38) 3 (41)
CV events – FOURIER
Eligible - incidence rate 2.6/100 pers.yr
Ineligible - incidence rate 2.5/100 pers.yr
RR 1.04 (0.81-1.32)
Patients at risk (cumulative events)
450 (0) 307 (39) 183 (79) 79 (91) 17 (99)
852 (0) 619 (86) 402 (155) 190 (194) 52 (216)
CV events – REDUCE-IT
Eligible - incidence rate 3.0/100 pers.yr
Ineligible - incidence rate 2.5/100 pers.yr
RR 1.19 (0.81-1.75)
Patients at risk (cumulative events)
1199 (0) 851 (112) 539 (209) 249 (258) 66 (286)
103 (0) 75 (13) 46 (25) 20 (27) 3 (29)
CV events – CLEAR Outcomes
Eligible - incidence rate 2.2/100 pers.yr
Ineligible - incidence rate 2.7/100 pers.yr
RR 0.83 (0.64-1.08)
Patients at risk (cumulative events)
988 (0) 680 (95) 420 (185) 182 (219) 42 (240)
314 (0) 246 (30) 165 (49) 87 (66) 27 (75)
B
All-cause mortality – HPS
Eligible - incidence rate 3.8/100 pers.yr
Ineligible - incidence rate 4.3/100 pers.yr
RR 0.87 (0.71-1.07)
Patients at risk (cumulative events)
288 (0) 203 (41) 133 (81) 67 (103) 19 (118)
1014 (0) 779 (96) 520 (234) 242 (327) 62 (383)
All-cause mortality – TNT
Eligible - incidence rate 4.8/100 pers.yr
Ineligible - incidence rate 3.7/100 pers.yr
RR 1.29 (1.04-1.61)
Patients at risk (cumulative events)
1091 (0) 835 (100) 551 (247) 265 (347) 70 (406)
211 (0) 147 (37) 102 (68) 44 (83) 11 (95)
All-cause mortality – IMPROVE-IT
Eligible - incidence rate 5.5/100 pers.yr
Ineligible - incidence rate 3.8/100 pers.yr
RR 1.44 (1.08-1.93)
Patients at risk (cumulative events)
1204 (0) 913 (115) 606 (277) 290 (382) 78 (450)
98 (0) 69 (22) 47 (38) 19 (48) 3 (51)
All-cause mortality – FOURIER
Eligible - incidence rate 4.0/100 pers.yr
Ineligible - incidence rate 3.7/100 pers.yr
RR 1.06 (0.88-1.29)
Patients at risk (cumulative events)
450 (0) 329 (43) 211 (105) 96 (137) 19 (158)
852 (0) 653 (95) 442 (210) 213 (293) 63 (343)
All-cause mortality – REDUCE-IT
Eligible - incidence rate 3.6/100 pers.yr
Ineligible - incidence rate 3.9/100 pers.yr
RR 0.92 (0.66-1.29)
Patients at risk (cumulative events)
1199 (0) 902 (127) 602 (290) 287 (398) 78 (464)
103 (0) 80 (10) 51 (25) 22 (32) 3 (37)
All-cause mortality – CLEAR Outcomes
Eligible - incidence rate 3.7/100 pers.yr
Ineligible - incidence rate 4.0/100 pers.yr
RR 0.92 (0.75-1.12)
Patients at risk (cumulative events)
988 (0) 723 (107) 472 (244) 206 (325) 48 (373)
314 (0) 259 (30) 181 (71) 103 (105) 33 (128)
Cumulative incidence
Follow−up time (years)

Supplementary Figure 4. Cumulative incidence of recurrent CV events (A) and all-cause mortality (B) in trial-eligible and trial-ineligible UCC-SMART patients with peripheral artery disease.

Rate ratios are presented with 95% confidence intervals for trial-eligible patients compared to trial-ineligible patients. UCC-SMART = Utrecht Cardiovascular Cohort–Second Manifestations of ARTerial disease; CV = cardiovascular; pers.yr = person-years; RR = rate ratio; HPS = Heart Protection Study; TNT = Treating to New Targets trial; IMPROVE-IT = IMProved Reduction of Outcomes: Vytorin Efficacy Intervention Trial; FOURIER = Further Cardiovascular Outcomes Research with PCSK9 Inhibition in Subjects with Elevated Risk; PCSK9 = proprotein convertase subtilisin/kexin type 9; REDUCE-IT = Reduction of Cardiovascular Events with Icosapent Ethyl–Intervention Trial; CLEAR Outcomes = Cholesterol Lowering via Bempedoic Acid, an ACL-Inhibiting Regimen Outcomes trial.

Supplementary Table 5. Sensitivity analysis of the proportion of UCC-SMART patients meeting trial eligibility criteria from 2010 onward.

Trial	Inclusion year 2000-2023 (full analysis)	Inclusion year 2010-2023 (sensitivity analysis)
**Coronary artery disease patients**	**N = 5,673**	**N = 2,830**
HPS	4,843 (85%)	2,438 (86%)
TNT	4,123 (73%)	2,119 (75%)
IMPROVE-IT	1,902 (34%)	792 (28%)
FOURIER	2,067 (36%)	1,020 (36%)
REDUCE-IT	818 (14%)	432 (15%)
CLEAR Outcomes	490 (9%)	161 (6%)
**Cerebrovascular disease patients**	**N = 2,493**	**N = 1,232**
SPARCL	936 (38%)	472 (38%)
TST	1,045 (42%)	478 (39%)
IMPROVE-IT	164 (7%)	72 (6%)
FOURIER	764 (31%)	388 (31%)
REDUCE-IT	190 (8%)	108 (9%)
CLEAR Outcomes	255 (10%)	115 (9%)
**Peripheral artery disease patients**	**N = 1,302**	**N = 470**
HPS	1,014 (78%)	369 (79%)
TNT	211 (16%)	77 (16%)
IMPROVE-IT	98 (8%)	34 (7%)
FOURIER	852 (65%)	277 (59%)
REDUCE-IT	103 (8%)	53 (11%)
CLEAR Outcomes	314 (24%)	66 (14%)

Data represent the number (%) of trial-eligible patients. UCC-SMART = Utrecht Cardiovascular Cohort–Second Manifestations of ARTerial disease; N = number; HPS = Heart Protection Study; TNT = Treating to New Targets trial; IMPROVE-IT = IMProved Reduction of Outcomes: Vytorin Efficacy Internation Trial; FOURIER = Further Cardiovascular Outcomes Research with PCSK9 Inhibition in Subjects with Elevated Risk; PCSK9 = proprotein convertase subtilisin/kexin type 9; REDUCE-IT = Reduction of Cardiovascular Events with Icosapent Ethyl–Intervention Trial; CLEAR Outcomes = Cholesterol Lowering via Bempedoic Acid, an ACL-Inhibiting Regimen Outcomes trial; SPARCL = Stroke Prevention by Aggressive Reduction in Cholesterol Levels trial; TST = Treat Stroke to Target trial.

Supplementary Table 6. Sensitivity analysis of rate ratios for recurrent CV events and all-cause mortality for trial-eligible compared to trial-ineligible UCC-SMART patients from 2010 onward.

Trial	Recurrent CV events		All-cause mortality	
	Inclusion year 2000–2023 (full analysis)	Inclusion year 2010–2023 (sensitivity analysis)	Inclusion year 2000–2023 (full analysis)	Inclusion year 2010–2023 (sensitivity analysis)
**Coronary artery disease patients**	**N = 5,673**	**N = 2,830**	**N = 5,673**	**N = 2,830**
HPS	0.81 (0.69–0.95)	0.75 (0.54–1.05)	0.80 (0.70–0.93)	0.67 (0.50–0.92)
TNT	0.70 (0.62–0.79)	0.57 (0.45–0.74)	0.60 (0.54–0.67)	0.47 (0.37–0.60)
IMPROVE-IT	1.00 (0.88–1.13)	1.05 (0.82–1.35)	1.06 (0.95–1.18)	1.13 (0.88–1.44)
FOURIER	1.55 (1.37–1.75)	1.37 (1.07–1.74)	1.49 (1.33–1.66)	1.33 (1.05–1.68)
REDUCE-IT	0.93 (0.78–1.11)	1.01 (0.72–1.41)	0.92 (0.78–1.08)	0.89 (0.63–1.25)
CLEAR Outcomes	1.26 (1.05–1.51)	1.54 (0.99–2.38)	1.29 (1.09–1.53)	1.40 (0.91–2.17)
**Cerebrovascular disease patients**	**N = 2,493**	**N = 1,232**	**N = 2,493**	**N = 1,232**
SPARCL	0.68 (0.56–0.83)	0.57 (0.39–0.85)	0.70 (0.59–0.82)	0.43 (0.29–0.63)
TST	1.68 (1.40–2.01)	1.49 (1.04–2.13)	1.42 (1.22–1.66)	1.30 (0.93–1.82)
IMPROVE-IT	2.12 (1.60–2.81)	2.42 (1.41–4.17)	1.94 (1.52–2.48)	2.38 (1.45–3.91)
FOURIER	1.93 (1.60–2.32)	1.94 (1.35–2.79)	1.59 (1.36–1.86)	1.72 (1.23–2.40)
REDUCE-IT	1.45 (1.07–1.97)	1.95 (1.17–3.26)	1.29 (0.98–1.69)	1.89 (1.18–3.04)
CLEAR Outcomes	1.29 (0.98–1.69)	0.41 (0.17–1.01)	1.21 (0.96–1.53)	0.53 (0.25–1.14)
**Peripheral artery disease patients**	**N = 1,302**	**N = 470**	**N = 1,302**	**N = 470**
HPS	0.99 (0.75–1.30)	0.84 (0.45–1.56)	0.87 (0.71–1.07)	0.82 (0.50–1.33)
TNT	1.66 (1.27–2.16)	2.15 (1.23–3.79)	1.29 (1.04–1.61)	1.62 (1.00–2.62)
IMPROVE-IT	1.94 (1.40–2.70)	3.15 (1.67–5.94)	1.44 (1.08–1.93)	2.00 (1.11–3.60)
FOURIER	1.04 (0.81–1.32)	1.22 (0.71–2.10)	1.06 (0.88–1.29)	0.99 (0.65–1.51)
REDUCE-IT	1.19 (0.81–1.75)	2.10 (1.13–3.89)	0.92 (0.66–1.29)	1.32 (0.74–2.33)
CLEAR Outcomes	0.83 (0.64–1.08)	0.37 (0.13–1.03)	0.92 (0.75–1.12)	0.64 (0.33–1.24)

Rate ratios are presented with 95% confidence intervals. UCC-SMART = Utrecht Cardiovascular Cohort–Second Manifestations of ARTerial disease; CV = cardiovascular; N = number; HPS = Heart Protection Study; TNT = Treating to New Targets trial; IMPROVE-IT = IMProved Reduction of Outcomes: Vytorin Efficacy Internation Trial; FOURIER = Further Cardiovascular Outcomes Research with PCSK9 Inhibition in Subjects with Elevated Risk; PCSK9 = proprotein convertase subtilisin/kexin type 9; REDUCE-IT = Reduction of Cardiovascular Events with Icosapent Ethyl–Intervention Trial; CLEAR Outcomes = Cholesterol Lowering via Bempedoic Acid, an ACL-Inhibiting Regimen Outcomes trial; SPARCL = Stroke Prevention by Aggressive Reduction in Cholesterol Levels trial; TST = Treat Stroke to Target trial.
